# Deciphering the therapeutic mechanisms of Qingfei Litan prescription in acute lung injury: an integrated framework of UHPLC–MS/MS, systems pharmacology, machine learning, and *in vivo* validation

**DOI:** 10.3389/fcimb.2026.1898960

**Published:** 2026-09-03

**Authors:** Wenyu Wu, Shaofeng Zhan, Kai Wang

**Affiliations:** 1 The First Affiliated Hospital of Guangzhou University of Chinese Medicine, Guangzhou, China; 2 The First Clinical Medical School of Guangzhou University of Chinese Medicine, Guangzhou, China; 3 Guangdong Clinical Research Academy of Chinese Medicine, Guangzhou, China

**Keywords:** acute lung injury, mechanism, molecular docking, network pharmacology, Qingfei Litan prescription

## Abstract

**Background:**

Acute lung injury (ALI) is primarily defined by hyper-inflammation and the breakdown of the alveolar-capillary barrier, frequently culminating in refractory respiratory failure. Current clinical interventions—predominantly glucocorticoids and supportive therapies—often yield suboptimal outcomes and are associated with significant side effects. Qingfei Litan Prescription (QFLTP), a traditional Chinese medical prescription indicated for inflammatory respiratory disorders, exhibits significant therapeutic potential; however, the precise molecular mechanisms driving its protective effects are currently under-investigated.

**Methods:**

Qualitative detection of the QFLTP ingredients was accomplished via UHPLC-MS/MS analysis. Active ingredients and their potential targets were screened using online prediction tools and traditional Chinese medicine databases, Concurrently, genes associated with ALI were retrieved via GeneCards, DisGeNET, and OMIM repositories. The intersection of these target sets underwent rigorous investigation, encompassing protein-protein interaction (PPI) mapping, alongside Gene Ontology (GO) and KEGG pathway enrichment evaluations, and multiple data-driven algorithms to identify hub genes. Computational modeling and simulation methods were performed to evaluate ligand-target binding. An LPS-induced ALI mouse model was established for *in vivo* validation. Histopathological examination, bronchoalveolar lavage fluid (BALF) protein quantification, inflammatory cytokine assays, arterial blood gas analysis, immunohistochemistry, immunofluorescence, Western blot, and qRT-PCR served to evaluate the therapeutic potential of QFLTP.

**Results:**

A total of 47 chemical constituents and 298 overlapping therapeutic targets were identified. Integrated computational and data-driven analyses highlighted POLB, FGF1, and ARG1 as potential hub genes involved in QFLTP-mediated protection against ALI. GO and KEGG enrichment analyses revealed that pharmacological restorative capacity of QFLTP activity demonstrated a robust functional linkage to inflammatory responses, PI3K-Akt signaling, VEGF signaling, and immune-related pathways. *In vivo* trials revealed that QFLTP administration markedly mitigated pulmonary histopathological impairment while simultaneously suppressing the secretion of pro-inflammatory mediators and pulmonary edema, improving lung function and arterial blood gas parameters, and promoted the recovery of junctional protein expression of ZO-1, ZO-2, and VE-cadherin in lung tissues. In addition, qRT-PCR profiling revealed that QFLTP treatment effectively normalized the aberrant mRNA levels of POLB, FGF1, and ARG1 in the lung tissues of the ALI cohort.

**Conclusion:**

QFLTP exerts protective effects against ALI via its multi-component, multi-target, and multi-pathway mechanisms, potentially by suppressing inflammatory responses, restoring pulmonary barrier integrity, and regulating the expression of hub genes including POLB, FGF1, and ARG1.

## 1 Introduction

Acute lung injury (ALI) represents a critical acute hypoxic respiratory failure syndrome triggered by diverse internal and external insults, resulting in damage of the alveolar-capillary barrier. Its key pathological features include alveolar edema, massive inflammatory cell infiltration, and markedly increased vascular permeability. ALI involves complex pathophysiological processes including uncontrolled immune activation, oxidative stress, hypoxia, amplified inflammatory cascades, cytokine storms, and electrolyte disturbances (Sapru et al., 2015; Thompson et al., 2017). Without timely and effective treatment, ALI frequently progresses to the more debilitating condition of acute respiratory distress syndrome (ARDS) (Matthay and Zimmerman, 2005; Sapoznikov et al., 2019), with a mortality rate of 35%–55% (Hayes et al., 2012; Levitt et al., 2013). Although current therapeutic approaches—including mechanical ventilation, oxygen support, glucocorticoids, and antibiotics—have improved, overall mortality remains unacceptably high, accompanied by considerable adverse effects and limited long-term benefits (Fragoso et al., 2017; Kligerman, 2024; Swenson and Swenson, 2021). Thus, there is a compelling need to discover safer and more targeted therapeutic approaches with clear mechanisms of action.

Traditional Chinese Medicine (TCM) represents a comprehensive medical system that has evolved over more than two millennia and is grounded in unique physiological and pathological perspectives. From the perspective of TCM, ALI is primarily attributed to “heat toxin,” “phlegm retention,” and “blood stasis,” with phlegm and stasis being central contributors (Ding et al., 2020). Formulas designed to clear heat and resolve phlegm have been widely applied clinically for respiratory disorders such as cough, pneumonia, and bronchitis. For instance, *Scutellaria baicalensis* is commonly used to treat lung infections by clearing heat, purging fire, detoxifying, and resolving phlegm (Zhao et al., 2019),whereas *Nervilia fordii* is frequently utilized in clinical practice to alleviate respiratory ailments, including persistent coughing, pharyngeal edema, bronchitis, and pneumonia (Qiu et al., 2013; Wen et al., 2021).

Qingfei Litan Prescription (QFLTP) is a modified formula based on the classical prescriptions Ma Xing Shi Gan Tang and Qian Jin Reed Stem Decoction from *Shang Han Lun* and *Qian Jin Fang*. It is traditionally employed to clear heat, resolve phlegm, regulate lung function, and relieve cough (Guo et al., 2021). According to TCM compatibility theory, Ma Xing Shi Gan Tang emphasizes clearing lung heat and relieving cough and asthma, while Qian Jin Reed Stem Decoction focuses on clearing lung heat and resolving phlegm (Jiang et al., 2024; Liu et al., 2022). Based on these therapeutic principles, QFLTP integrates herbs with complementary functions of clearing heat, eliminating phlegm, regulating lung qi, and protecting pulmonary function, reflecting the characteristic multi-herb synergy of TCM formulas. QFLTP consists of ten medicinal herbs: *Nervilia fordii* (Hance) Schltr. (Qingtiankui), Gypsum Fibrosum (Shengshigao), *Trichosanthes kirilowii* Maxim. (Gualoupi), *Scutellaria baicalensis* Georgi (Huangqin), *Fritillaria thunbergii* Miq. (Zhebeimu), *Houttuynia cordata* Thunb. (Yuxingcao), *Phragmites australis* (Cav.) Trin. ex Steud. (Lugen), *Prunus armeniaca* L. (Kuxingren), *Platycodon grandiflorus* A.DC. (Jiegeng), and *Glycyrrhiza uralensis* Fisch. ex DC. (Gancao) (**Table 1**). Among them, *Scutellaria baicalensis* and Gypsum Fibrosum mainly contribute to clearing lung heat, while *Trichosanthes kirilowii*, *Fritillaria thunbergii*, and *Houttuynia cordata* assist in resolving phlegm and alleviating inflammation. Platycodon grandiflorus, Prunus armeniaca, and Glycyrrhiza uralensis contribute to regulating lung qi, relieving cough, and harmonizing the formula (Yao et al., 2021; Zhao et al., 2016). Each herb has been extensively documented in classical materia medica for clearing heat, resolving phlegm, and addressing pulmonary conditions.

**Table 1:** The pharmaceutical composition of QFLTP.

Chinese name	Vegetal product	Latin name	Family	Part used	Dose (g)
Qingtiankui (QTK)	Asparagales	*Nervilia fordii* (Hance) Schltr.	Orchidaceae	Leaf	10
Shengshigao (SSG)	/	Gypsum Fibrosum	Sulfate gypsum group minerals	Gypsum	30
Gualoupi (GLP)	trichosanthes kirilowii maxim or trichosanthes rosthornii harms	*Trichosanthes kirilowii* Maxim.	Cucurbitaceae	Pericarp	15
Huangqin (HQ)	Scutellaria Root	*Scutellaria baicalensis* Georgi	Lamiaceae	Root	15
Zhebeimu (ZBM)	fritillaria thunbergii	*Fritillaria thunbergii* Miq.	Liliaceae	Bulb	15
Yuxingcao (YXC)	Houttuynia cordata	*Houttuynia cordata* Thunb.	Saururaceae	Aerial part	20
Lugen (LG)	Phragmitis Rhizoma	*Phragmites australis (Cav.)* Trin. ex Steud.	Gramineae	Rhizome	15
Kuxingren (KXR)	armeniacae semen amarum	*Prunus armeniaca* L.	Rosaceae (rose family)	Seed	10
Jiegeng (JG)	Platycodon grandiflorum (Jacq.)A.DC	*Platycodon grandiflorus* A.DC.	Campanulaceae	Root	15
Gancao (GC)	Licorice, licorice with swollen fruit or licorice with light fruit	*Glycyrrhiza uralensis* Fisch. ex DC.	Leguminosae	Root and rhizome	10

Our previous clinical observations and animal experiments showed that QFLTP effectively improved symptoms in pneumonia patients and mitigated LPS-induced ALI in mice, partly through suppression of oxidative stress (Guan-bin et al., 2014; Kai et al., 2016). However, the molecular mechanisms, including active compounds, target genes, signaling pathways, and compound-target interactions, are still not fully understood.

Due to the multi-component and multi-target nature of TCM formulae, traditional pharmacological approaches are frequently insufficient for completely clarifying their activities (Ma et al., 2016).Network pharmacology provides a robust systems-level framework for delineating the intricate web of associations linking bioactive compounds, molecular targets, and biological signaling cascades within TCM (Jin et al., 2021; Niu et al., 2022; Wu et al., 2024; 2022). However, computational predictions alone are insufficient to establish definitive causal relationships. Therefore, combining multiple computational prediction methods with rigorous *in vivo* experimental validation is essential to strengthen the reliability and clinical translational value of the mechanistic findings (Hopkins, 2008; Jiao et al., 2021; Li and Zhang, 2013).

In the present study, we hypothesized that QFLTP protects against ALI mainly by coordinately suppressing inflammatory responses and restoring endothelial/epithelial barrier integrity through multiple key regulatory nodes. To test this hypothesis, we employed an integrative strategy involving computational target prediction, advanced data-driven analyses, molecular docking, molecular dynamics simulations, and LPS-induced ALI mouse model experiments to identify core bioactive ingredients and key regulatory genes. These predictions were subsequently validated in animal experiments. This comprehensive approach aims to bridge traditional ethnopharmacological knowledge with modern molecular evidence and elucidate the underlying molecular framework guiding the therapeutic administration of QFLTP for ALI.

## 2 Materials and methods

### 2.1 Identification of chemical constituents in QFLTP

#### 2.1.1 Preparation of QFLTP

QFLTP was prepared from the following ten herbs with specified dosages: *Nervilia fordii* (Hance) Schltr. (Qingtiankui, 10 g), Gypsum Fibrosum (Shengshigao, 30 g)*, Trichosanthes kirilowii* Maxim. (Gualoupi, 15 g)*, Scutellaria baicalensis* Georgi. (Huangqin, 15 g), *Fritillaria thunbergii* Miq. (Zhebeimu, 15 g), *Houttuynia cordata* Thunb. (Yuxingcao, 20 g), *Phragmites australis (Cav.)* Trin. ex Steud. (Lugen, 15 g), *Prunus armeniaca* L. (Kuxingren, 10 g), *Platycodon grandiflorus* A.DC. (Jiegeng, 15 g)*, and Glycyrrhiza uralensis* Fisch. ex DC. (Gancao, 10 g). The total amount of crude herbs was 155 g. The herbs were soaked in eight-fold volume warm water for 0.5 h and decocted for 4 h. The residues were further decocted twice with six-fold volume of water for 2 h each time. The three decoctions were combined, filtered, and concentrated to obtain the final QFLTP decoction. The final preparation was not lyophilized and was directly used for animal administration. The decoction was stored at 4 °C until use. The dosage of QFLTP was determined based on the standard interspecies dose conversion method using body surface area normalization (Ji-Han et al., 2004). The equivalent mouse dose was calculated according to the adult human dose of QFLTP (57 g crude herbs/day) 0.147 g crude herbs per 20 g mouse (7.35 g/kg), which was defined as the medium dose. The high- and low-dose groups were established at 2-fold (0.294 g/mouse) and 0.5-fold (0.0735 g/mouse) of the medium dose, respectively.

#### 2.1.2 UHPLC-MS/MS analysis of QFLTP

The chemical profile of QFLTP was delineated through high-performance liquid chromatography coupled with mass spectrometry, utilizing an Ultimate 3000 platform (Thermo Fisher Scientific, USA). The QFLTP decoction was mixed with methanol, subjected to ultrasonic extraction, and subsequently centrifuged and filtered prior to analysis. Chromatographic resolution was achieved using a C18 stationary phase, using a gradient elution profile of acetonitrile and 0.1% formic acid. Both positive and negative polarity modes were enabled for mass spectrometric acquisition. Compound verification relied upon aligning retention characteristics, precursor ions, and fragmentation patterns with established literature, with subsequent data curation via Xcalibur software.

#### 2.1.3 Screening of active ingredients and target prediction

Molecular targets were predicted by uploading canonical SMILES structures of the identified compounds into the SwissTargetPrediction portal (human species, probability score ≥ 0.1). We standardized protein identifiers into gene symbols using reviewed records from the UniProt database. Furthermore, TCMSP-derived compounds were subjected to drug-likeness and oral bioavailability filtering (DL ≥ 0.18; OB ≥ 30%). These curated compounds and their corresponding targets were *subsequently synthesized into the analytical network.*

### 2.2 Network pharmacology analysis

#### 2.2.1 Construction of the herb–compound–target network

The herb-compound-target network was built using Cytoscape 3.8.2, which imported intersecting targets between QFLTP active components and ALI-related genes. Topological parameters were examined using the NetworkAnalyzer plugin. Compounds with high node degree *values were identified as important bioactive components.*

#### 2.2.2 Collection of ALI-related targets

ALI-associated targets were retrieved by searching for “acute lung injury” in GeneCards, DisGeNET, and OMIM databases. Duplicates were removed, and only targets with relevance scores above the median were retained.

#### 2.2.3 Identification of therapeutic targets of QFLTP for ALI

A Venn diagram and Excel were used to identify overlaps between QFLTP-predicted and ALI-related targets. These common targets were identified as possible QFLTP treatment targets for ALI.

#### 2.2.4 Construction of the PPI network and core target identification

The shared targets were submitted to the STRING database (v11.5, confidence score ≥ 0.7, Homo sapiens) to build the PPI network (Szklarczyk et al., 2019). To prioritize critical targets within the network, we performed a topological analysis, ranking proteins based on their degree distribution, betweenness centrality, and closeness centrality scores (Xia et al., 2020).

#### 2.2.5 Functional enrichment analysis

To explore the functional significance of the identified targets, we performed Gene Ontology (GO) and KEGG enrichment analyses via the Metascape platform. The investigation spanned biological processes (BP), cellular components (CC), molecular functions (MF), and pathway signaling circuits. Terms with a Benjamini-Hochberg adjusted *P* value below 0.05 were considered statistically significant. The enrichment results were visualized using multiple online bioinformatics tools.

#### 2.2.6 Identification of hub genes using machine learning

To refine the target selection, we employed a tripartite machine learning framework comprising random forest (RF), support vector machine-recursive feature elimination (SVM-RFE), and LASSO regression. The transcriptomic dataset GSE10474 from the Gene Expression Omnibus (GEO) database was used for hub gene screening and subsequent validation. The LASSO model was optimized via 10-fold cross-validation leveraging the R glmnet package (version 4.1.2). Parallel to this, the SVM-RFE and RF models were implemented utilizing the e1071 and randomForest R packages, respectively. Genes detected by all three algorithms were defined as core hub genes. These hub genes were subsequently validated through differential expression analysis and correlation analysis in the GSE10474 ALI dataset.

#### 2.2.7 Gene set enrichment analysis

Based on the expression profiles from the GSE10474 dataset, samples were partitioned into distinct high- and low-expression clusters according to the median expression value of each hub gene. Functional enrichment was then examined using the clusterProfiler framework, setting the significance criterion at an adjusted *P* value below 0.05.

#### 2.2.8 Immune infiltration analysis

The proportions of immune cells were evaluated using CIBERSORT based on the GSE10474 dataset (Newman et al., 2015). To characterize the relationship between hub gene expression and local immune landscape, we applied Spearman’s rank correlation analysis. Furthermore, the ESTIMATE algorithm was deployed to quantify the respective immune and stromal scores within the tissue samples.

#### 2.2.9 Molecular docking

The interaction potential between active molecules and hub proteins was interrogated via docking calculations. Using protein models from RCSB PDB and ligand formats from PubChem, we prepared the system by removing solvent molecules and protonating the protein structures. Binding simulations were conducted in AutoDock Vina, and the minimum energy conformations were selected to illustrate potential binding interfaces using PyMOL.

#### 2.2.10 Molecular dynamics simulation

The stability of the ligand-protein binding poses was further verified 100 ns atomistic molecular dynamics simulations. Force field assignment employed FF14SB and GAFF for proteins and ligands, respectively. Systems were immersed in a TIP3P water bath subjected to periodic boundary conditions. Following a rigorous protocol of energy minimization, heating, and equilibration, 100 ns production simulations were carried out within the NPT ensemble. Trajectory data were processed using CPPTRAJ to extract relevant structural statistics.

### 2.3 Experimental validation

#### 2.3.1 Experimental animals

Male C57BL/6 mice (6–8 weeks old; 20–25 g) were sourced from Jinan Ponyue Laboratory Animal Breeding Co., Ltd. and housed under specific pathogen-free conditions. The experimental protocol received formal approval from the Institutional Animal Care and Use Committee (IACUC-2024003PD-01M-P), and all procedures were performed in strict alignment with national guidelines governing laboratory animal welfare.

#### 2.3.2 ALI induction, grouping, and drug administration

Following a seven-day acclimation period, mice were randomly assigned into six groups (n = 6 per group): healthy control group, ALI model group, dexamethasone group, and three QFLTP treatment groups (low-, medium-, and high-dose groups). Except for the control group, ALI was induced by intratracheal administration of lipopolysaccharide (LPS, 5 mg/kg in 50 μL saline) under isoflurane anesthesia. The LPS dose of 5 mg/kg was selected to induce a reproducible ALI phenotype characterized by pulmonary inflammation, alveolar-capillary barrier disruption, pulmonary edema, and inflammatory cytokine release. After LPS challenge, QFLTP administration was initiated and continued once daily for seven consecutive days. Mice were euthanized 24 h after the final administration for subsequent analyses.

The QFLTP doses were determined using the standard interspecies dose conversion method based on body surface area normalization (Ji-Han et al., 2004). Briefly, the clinical dose of QFLTP (57 g crude herbs/day for adults) was converted into the corresponding mouse dose of 0.147 g crude herbs per 20 g mouse (7.35 g/kg), which was defined as the medium dose. The high-dose and low-dose groups received 2-fold (0.294 g/mouse) and 0.5-fold (0.0735 g/mouse) of the medium dose, respectively.

QFLTP decoction was prepared from the prescribed herbs by soaking the crude drugs in eight-fold warm water for 0.5 h, followed by decoction at low heat for 4 h. The residues were subsequently extracted twice using six-fold water for 2 h each time. The three decoctions were combined, filtered through three layers of gauze, and concentrated in a water bath to obtain the final stock solution of 1.47 g crude herbs/mL. The medium-dose solution (0.735 g/mL) and low-dose solution (0.3675 g/mL) were prepared by 2-fold and 4-fold dilution of the stock solution, respectively. The prepared QFLTP solutions were cooled to room temperature and stored at 4 °C until use. All groups received oral gavage at a fixed volume of 0.2 mL/mouse once daily for seven consecutive days.

Dexamethasone (1.0 mg/kg) was administered intraperitoneally as a positive control, following commonly used protocols for ALI experimental studies. QFLTP was administered by oral gavage to mimic its clinical administration route, while the control and ALI model groups received equivalent volumes of vehicle solution by oral gavage. Twenty-four hours after the final administration, mice were euthanized, and lung tissues, BALF, and blood samples were collected for subsequent analyses, including pulmonary edema assessment, histological examination, and molecular analyses. The successful establishment of the ALI model was evaluated based on multiple quantitative parameters, including lung wet/dry (W/D) weight ratio, BALF protein concentration, arterial blood gas parameters, inflammatory cytokine levels, and histopathological changes.

#### 2.3.3 General observations

Respiratory rate, spontaneous activity, mental status, characteristics of urine and feces, mucosal color, ocular and nasal secretions, and mortality were monitored and recorded daily throughout the experimental period.

#### 2.3.4 Arterial blood gas analysis

Upon study termination, subjects were anesthetized via intraperitoneal injection of pentobarbital sodium (50 mg/kg). Arterial blood was subsequently harvested from the abdominal aorta. The immediate assessment of arterial PaO_2_, PaCO_2_, and pH levels was conducted using an i-STAT portable blood gas analyzer.

#### 2.3.5 Acquisition of BALF prior to protein quantification

Twenty-four hours after the final drug administration, BALF was collected. The right pulmonary lobe was tied off, and the left side underwent triple washing utilizing sterile saline containing a phosphate buffer. The recovered BALF underwent spin processing at 3000 rpm for 10 min at 4 °C, and the supernatant was collected for subsequent analysis. The total protein concentration in BALF was determined using a BCA protein assay kit as an indicator of alveolar-capillary barrier disruption and increased pulmonary vascular permeability, which are characteristic features of ALI. Total amount of protein in this BALF upper liquid was determined by employing a BCA protein evaluation kit.

#### 2.3.6 Measurement of lung wet/dry weight ratio

The lung wet/dry (W/D) weight ratio was measured to evaluate pulmonary edema. As pulmonary edema is a major pathological feature of ALI, the W/D ratio was used as a quantitative indicator of lung fluid accumulation and model establishment. After mice were euthanized, lung tissues were immediately collected and weighed to obtain the wet weight. The samples were then dried at 60 °C until a constant weight was achieved, and the dry weight was recorded. The W/D ratio was calculated by dividing the wet weight by the dry weight.

#### 2.3.7 Body weight measurement

Body weight was measured using a digital scale at 0, 12, 24, 72,120 and 168 h after LPS challenge, and then daily until study conclusion.

#### 2.3.8 Pulmonary performance analysis

At the end of the study, respiratory function was assessed in unrestrained conscious mice using a whole-body plethysmography system. Key parameters, including peak inspiratory flow (PIF), peak expiratory flow (PEF), and minute ventilation (MV), were recorded.

#### 2.3.9 Histological tissue analysis

After completion of the experiment, mice were anesthetized with pentobarbital sodium (50 mg/kg, i.p.). Lung tissues were collected, and samples from six randomly selected mice in each group were harvested, fixed in 10% formalin, embedded in paraffin, sectioned, and subjected to hematoxylin and eosin (H&E) staining. The severity of pulmonary injury was blindly evaluated according to the American Thoracic Society (ATS) guidelines using optical microscopy.

#### 2.3.10 ELISA protocol

At the end of the experiment, blood samples were collected from the orbital venous plexus under pentobarbital sodium anesthesia (50 mg/kg, i.p.). Samples were allowed to clot at 4 °C for 24 h and then centrifuged at 3000 rpm for 15 min. The obtained serum samples were stored at −80 °C until analysis. In addition, lung tissues were collected, homogenized in ice-cold phosphate-buffered saline (PBS), and centrifuged to obtain the tissue supernatants for cytokine determination. The levels of interleukin-6 (IL-6), interleukin-1β (IL-1β), and tumor necrosis factor-α (TNF-α) in both serum and lung tissue homogenates were measured using commercially available ELISA kits according to the manufacturer’s instructions.

#### 2.3.11 Immunohistochemical analysis

Paraffin-embedded lung sections were deparaffinized and rehydrated, followed by endogenous peroxidase blocking and heat-induced antigen retrieval. The sections were blocked at room temperature and incubated overnight at 4 °C with primary antibodies against ZO-1, ZO-2, and VE-cadherin. After washing with PBS, sections were incubated with appropriate secondary antibodies, visualized using DAB chromogen, counterstained with hematoxylin, dehydrated, cleared, and mounted. Images were captured and analyzed using a light microscope.

#### 2.3.12 Immunofluorescent examination

Paraffin-embedded lung sections were blocked with 1% bovine serum albumin (BSA) for 1 h at room temperature and subsequently incubated overnight at 4 °C with primary antibodies against ZO-1, ZO-2, and VE-cadherin. After PBS washing, sections were incubated with fluorophore-conjugated secondary antibodies for 1 h at room temperature. Nuclear staining was performed using 4′,6-diamidino-2-phenylindole (DAPI) for 10 min in the dark. Images were acquired using a fluorescence microscope.

#### 2.3.13 Western blotting analysis

Lung tissues (approximately 15 mg per sample) were homogenized for total protein extraction. Protein concentrations were determined using a BCA assay kit. Equal amounts of protein were denatured, separated by sodium dodecyl sulfate-polyacrylamide gel electrophoresis (SDS-PAGE), and transferred onto PVDF membranes. The membranes were blocked with 10% nonfat milk for 1 h and then incubated overnight at 4 °C with primary antibodies against ZO-1 (1:2000), ZO-2 (1:2000), VE-cadherin (1:2000), and GAPDH (1:5000). After washing with Tris-buffered saline containing Tween-20 (TBST), membranes were incubated with secondary antibodies (1:5000) for 1 h at room temperature. Protein bands were visualized and quantified using ImageJ software.

#### 2.3.14 qRT-PCR identification regarding target molecules

Total RNA was extracted from lung tissues using TRIzol reagent (Invitrogen, USA). RNA purity and concentration were assessed using a NanoDrop spectrophotometer (OD260/OD280 ratio between 1.8 and 2.0). One microgram of RNA was reverse-transcribed into cDNA using the PrimeScript RT reagent kit (Takara, Japan). Quantitative real-time PCR was performed using a StepOnePlus Real-Time PCR System (Applied Biosystems) with SYBR Green Master Mix. The amplification conditions were as follows: 95 °C for 30 s, followed by 40 cycles of 95 °C for 5 s and 60 °C for 30 s. Glyceraldehyde-3-phosphate dehydrogenase (GAPDH) was used as the internal control, and relative expression levels were calculated using the 2^−ΔΔCt method. Primer sequences are listed in **Table 2**.

**Table 2:** The sequences of the primers.

Gene	Forward primer	Reverse primer
POLB	TGCAGAGTCCAGTGGTGACATG	ATGAACCTTTTGTAACTGCTCCAC
FGF1	CCAAGGAAACGTCCACAGTCAG	ACGGCTGAAGACATCCTGTCTC
ARG1	CATTGGCTTGCGAGACGTAGAC	GCTGAAGGTCTCTTCCATCACC
GAPDH	AGGTCGGTGTGAACGGATTTG	TGTAGACCATGTAGTTGAGGTCA

### 2.4 Statistical evaluation methods

Reported findings are presented as mean ± standard deviation (SD). Statistical analyses were performed using GraphPad Prism 8.0. Data distribution and variance homogeneity were assessed before analysis of variance. Comparisons among multiple groups were performed using one-way analysis of variance (ANOVA) followed by Tukey’s *post-hoc* test. A two-tailed *P* value < 0.05 was considered statistically significant.

## 3 Results

### 3.1 UHPLC-MS/MS-based identification of major compounds in QFLTP

The chemical profile of QFLTP ethanol extracts was analyzed using UHPLC-MS/MS. Based on retention times, molecular ion signals, and comparison with published literature and the TCMSP database, a total of 47 chemical constituents were successfully identified. These compounds mainly consisted of 35 flavonoids, 4 alkaloids, 4 organic acids, 3 cyanogenic glycosides, and 1 coumarin. The identified compounds provided the basis for subsequent network pharmacology analyses (Diao et al., 2022).

### 3.2 Network pharmacology analysis

#### 3.2.1 Active constituent and likely molecular targets regarding QFLTP

**Figure 1:**
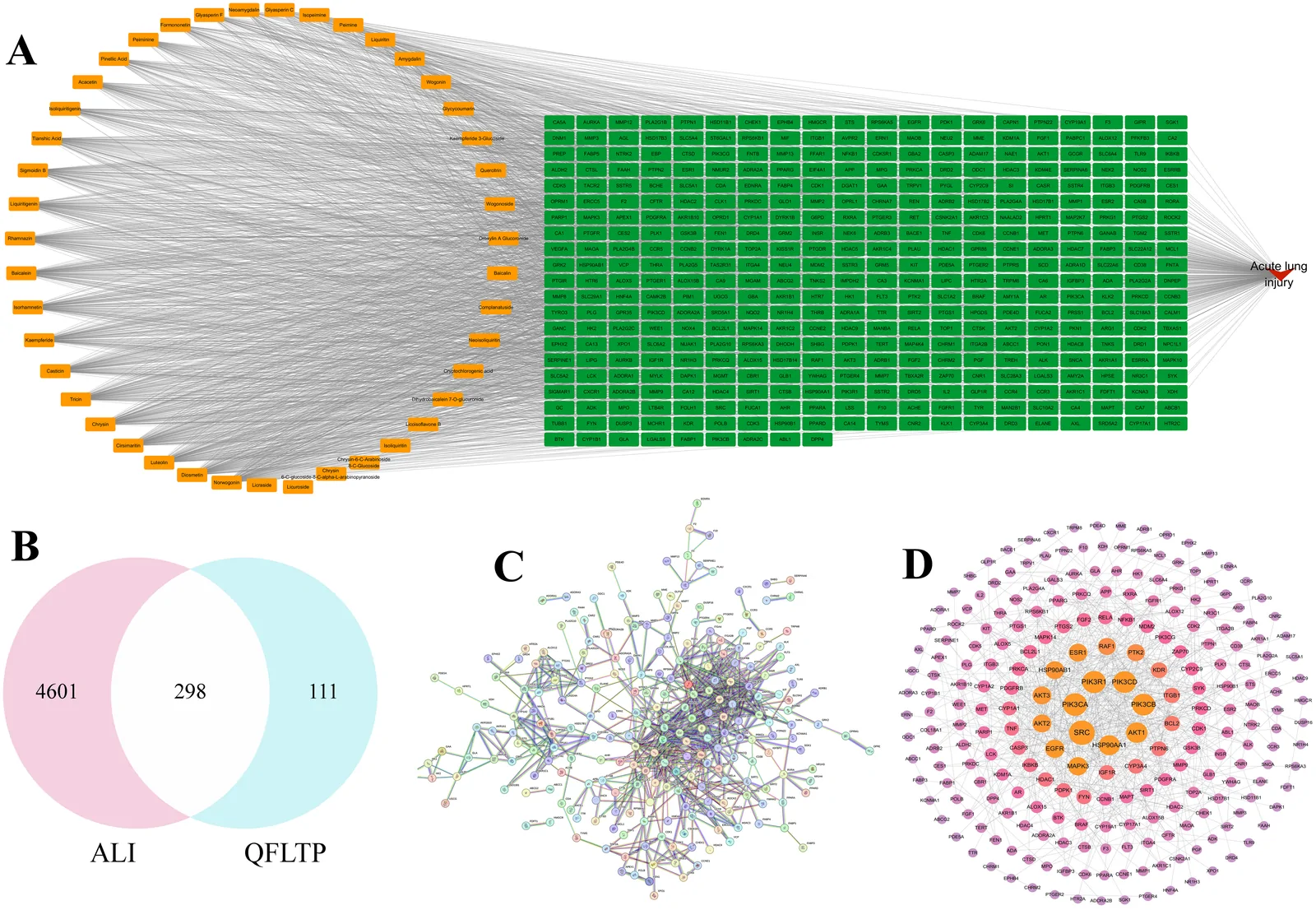
Core components and potential therapeutic targets of QFLTP in the treatment of ALI. **(A)** Herb–compound–target network. **(B)** intersection of QFLTP active ingredient targets and ALI related target. **(C)** PPI network construction. **(D)** Core network of protein interactions.

Utilizing the 47 identified compounds, a total of 409 candidate protein targets were predicted via the SwissTargetPrediction platform. Such entities transitioned inside Cytoscape 3.8.2 to construct the herb–compound–target interaction network, which possessed 453 vertices plus 2497 connections (**Figure 1A**). Topological analysis identified ten key bioactive compounds with high centrality values, including baicalein, casticin, chrysin, cirsimaritin, diosmetin, isorhamnetin, kaempferide, luteolin, norwogonin, and rhamnazin (**Table 3**).

**Table 3:** Key chemical components of QFLTP.

Compound name	Degree	Closeness centrality (CC)	Betweenness centrality (BC)
Baicalein	104	0.63040446	0.56708224
Casticin	104	0.40831075	0.02112841
Chrysin	104	0.40831075	0.01796292
Cirsimaritin	104	0.40831075	0.02250826
Diosmetin	104	0.40831075	0.02073137
Isorhamnetin	104	0.40831075	0.01817651
Kaempferide	104	0.40831075	0.01769138
Luteolin	104	0.40831075	0.01838017
Norwogonin	104	0.40831075	0.01894332
Rhamnazin	104	0.40831075	0.01785117

#### 3.2.2 ALI-elucidation regarding intersecting protein targets linking QFLTP plus ALI

ALI-associated targets were collected from the GeneCards, DisGeNET, and OMIM databases. After removing redundant entries, 4,899 unique candidate targets were obtained. Venn diagram analysis revealed 298 overlapping targets between QFLTP-related targets and ALI-associated genes (**Figure 1B**). These shared targets were considered potential therapeutic targets through which QFLTP may exert protective effects against ALI.

#### 3.2.3 PPI network assembly

The 298 overlapping targets were imported into the STRING database (v11.5, combined score ≥ 0.7) to construct the PPI network. Network visualization and topological analysis were performed using Cytoscape 3.8.2 (**Figures 1C, D**). Hub targets were identified based on key topological parameters, including degree centrality, betweenness centrality, and closeness centrality.

#### 3.2.4 GO and KEGG enrichment analysis

**Figure 2:**
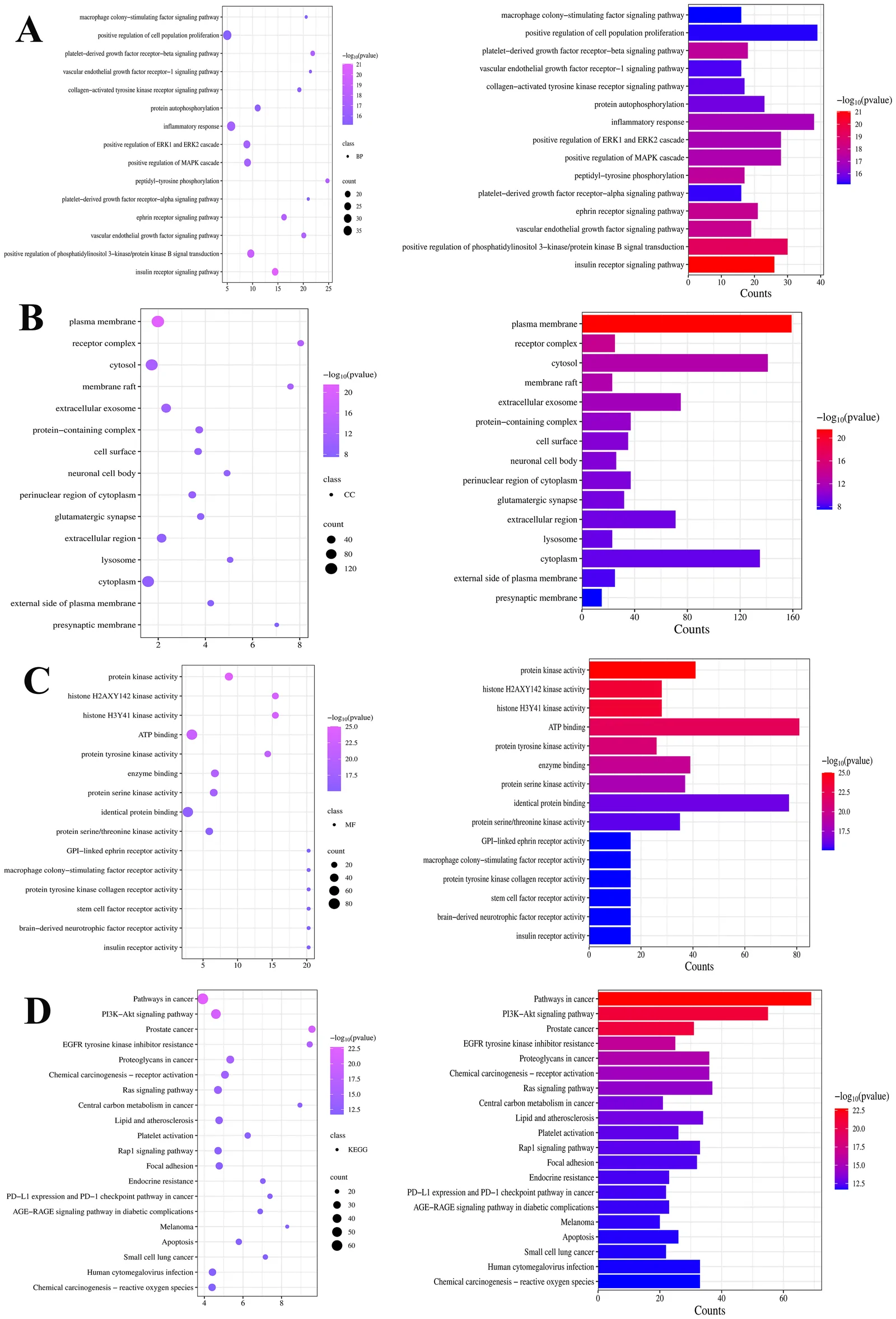
Functional enrichment analysis of overlapping targets for QFLTP in ALI treatment. **(A)** Biological process enrichment. **(B)** Cellular component enrichment. **(C)** Molecular function. **(D)** KEGG pathway enrichment.

Metascape-based GO enrichment analysis identified 1,305 significantly enriched functional categories. The major enriched terms were closely associated with inflammatory responses, protein phosphorylation, MAPK signaling pathways, PI3K-Akt signaling, and regulation of cellular homeostasis and proliferation (**Figures 2A–C**). KEGG pathway enrichment analysis identified 168 significantly enriched pathways, including PI3K-Akt, AGE-RAGE, and VEGF signaling pathways, as well as various immune-related networks (**Figure 2D**; **Supplementary Table 2**). These findings suggest that QFLTP may alleviate ALI through multi-target regulation and coordinated modulation of multiple signaling pathways.

#### 3.2.5 Identification of hub genes using machine learning

**Figure 3:**
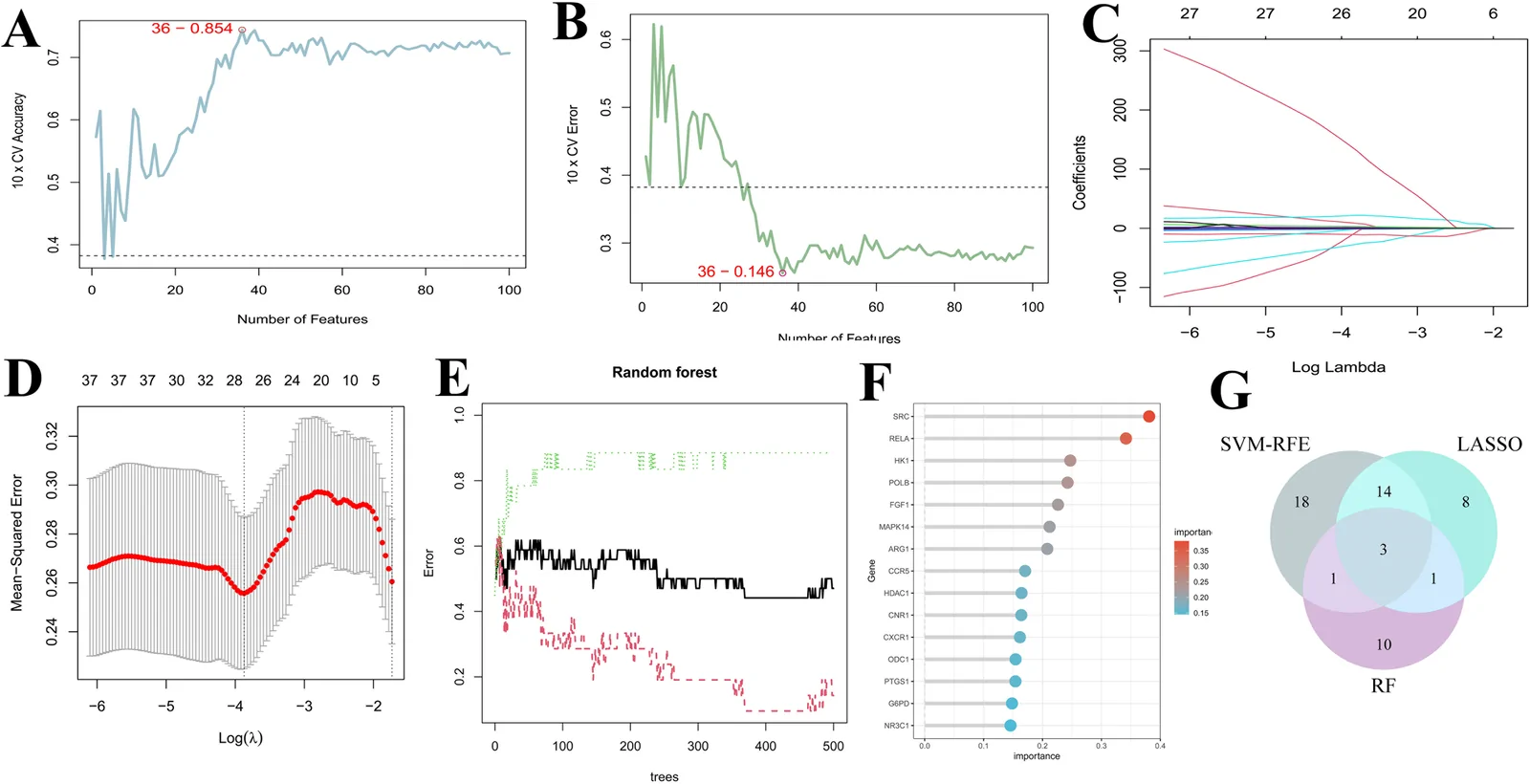
Identification of hub genes for QFLTP in ALI treatment using machine learning algorithms. **(A)** 10-fold cross-validation accuracy curve used to determine the optimal number of features. **(B)** Error rate curve demonstrating the minimization of classification error in selecting the most discriminative gene subset. **(C)** Coefficient profiles of potential genes plotted against the log(lambda) sequence. **(D)** Regularization path showing the optimal lambda value selected via cross-validation to prevent overfitting. **(E)** Error rate curve illustrating the stabilization of the model based on the number of decision trees. **(F)** Variable Importance in Projection (VIP) plot, ranking the genes by their contribution to the model’s predictive accuracy. **(G)** Identification of hub targets. Venn diagram showing the intersection of key genes selected by the three independent machine learning approaches (SVM-RFE, LASSO, and RF), confirming the robust identification of core targets.

Three machine learning algorithms, including SVM-RFE, LASSO regression, and random forest (RF), were applied to identify high-confidence hub genes from the 298 overlapping targets. The intersection of genes selected by all three methods yielded three hub genes: POLB, FGF1, and ARG1 (**Figure 3**). These genes were prioritized as key regulators in QFLTP-mediated protection against ALI.

#### 3.2.6 GSEA of the three hub genes

**Figure 4:**
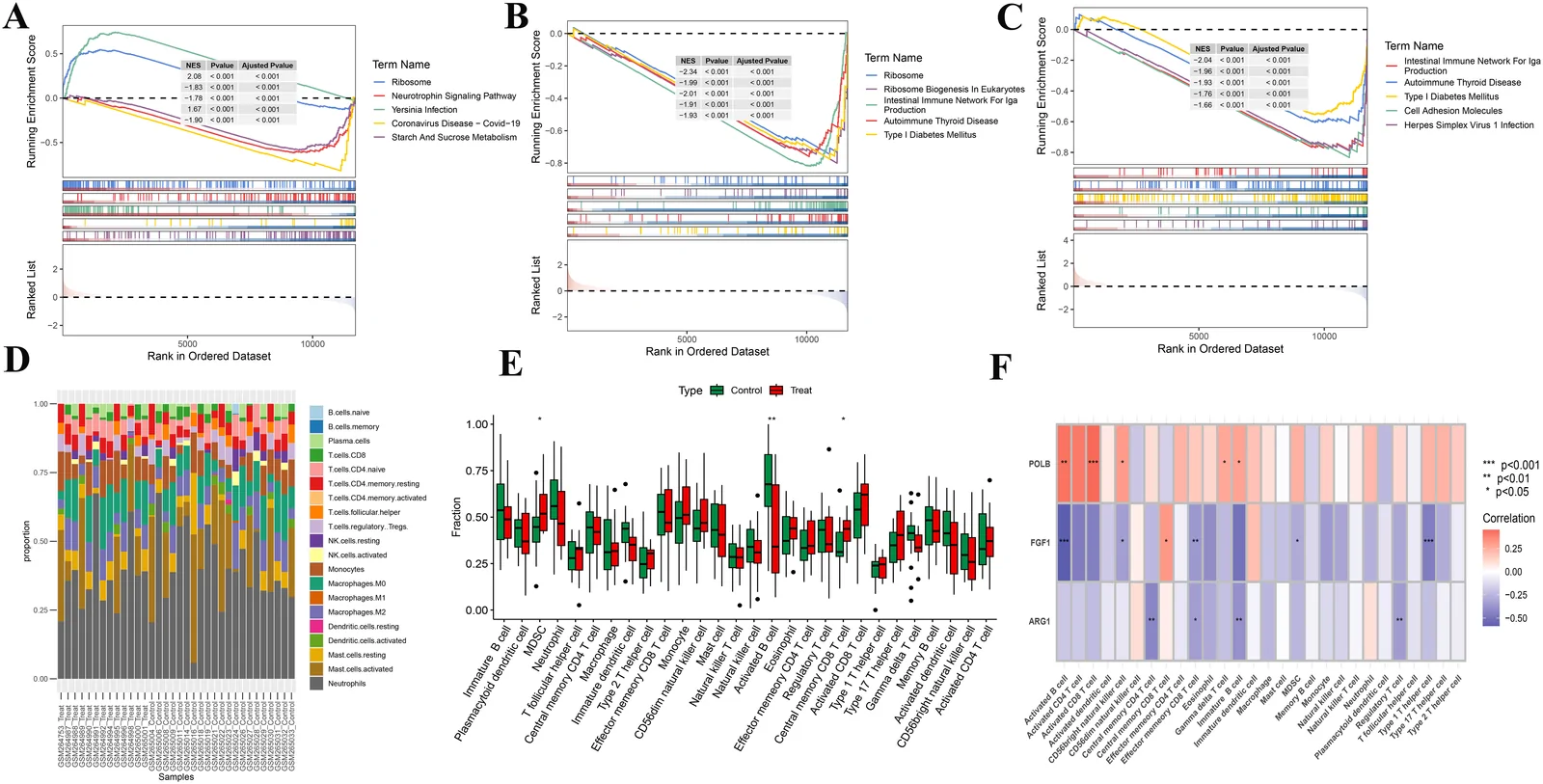
Functional enrichment, immune landscape, and hub gene-immune correlation analysis. **(A–C)** Gene Set Enrichment Analysis (GSEA). Functional enrichment profiles revealing significant pathway activity associated with translational regulation (e.g., ribosome, ribosome biogenesis), metabolic adaptation (e.g., starch and sucrose metabolism), and immune response/autoimmunity (e.g., Yersinia infection, COVID-19, autoimmune thyroid disease, type I diabetes mellitus, and cell adhesion molecules). Data are presented with Normalized Enrichment Scores (NES), nominal P-values, and adjusted P-values. **(D)** Immune cell composition. Relative proportions of 22 distinct immune cell subpopulations estimated via the CIBERSORT algorithm, illustrating the shift in the immune landscape between experimental groups. **(E)** Comparative immune abundance. Boxplots visualizing the differential infiltration levels of immune cell types between the control and ALI groups, identifying significantly altered populations in ALI lung tissues. **(F)** Correlation analysis. Heatmap depicting the Spearman/Pearson correlations between the expression of hub genes (POLB, FGF1, ARG1) and the levels of immune cell infiltration. Correlations are color-coded (red = positive, blue = negative), with asterisks denoting statistical significance (^*^P < 0.05, ^**^P < 0.01, ^***^P < 0.001).

Following stratification of the GSE10474 dataset into high- and low-expression groups based on the median expression level of each hub gene, GSEA was performed. The results showed distinct pathway associations: POLB was enriched in ribosome, neurotrophin signaling, and metabolic pathways; FGF1 was associated with ribosome biogenesis and immune-related processes; and ARG1 was associated with cell adhesion and viral infection pathways (**Figures 4A–C**). These findings suggest that the three hub genes play important regulatory roles in protein synthesis, immune responses, and inflammatory signaling during ALI progression.

#### 3.2.7 Immune infiltration analysis

CIBERSORT analysis of the GSE10474 dataset revealed significant alterations in immune cell infiltration in ALI lung tissues (**Figure 4D**). Specifically, the proportions of activated B cells, plasmacytoid dendritic cells, myeloid-derived suppressor cells (MDSCs), neutrophils, and activated CD8^+^ T cells were increased, whereas follicular helper T cells, monocytes, and natural killer (NK) cells were decreased (**Figure 4E**). Correlation analysis demonstrated that the expression levels of the hub genes were significantly associated with the infiltration of multiple immune cell populations (**Figure 4F**), suggesting that these genes may participate in immune regulation during ALI.

#### 3.2.8 Molecular docking

**Figure 5:**
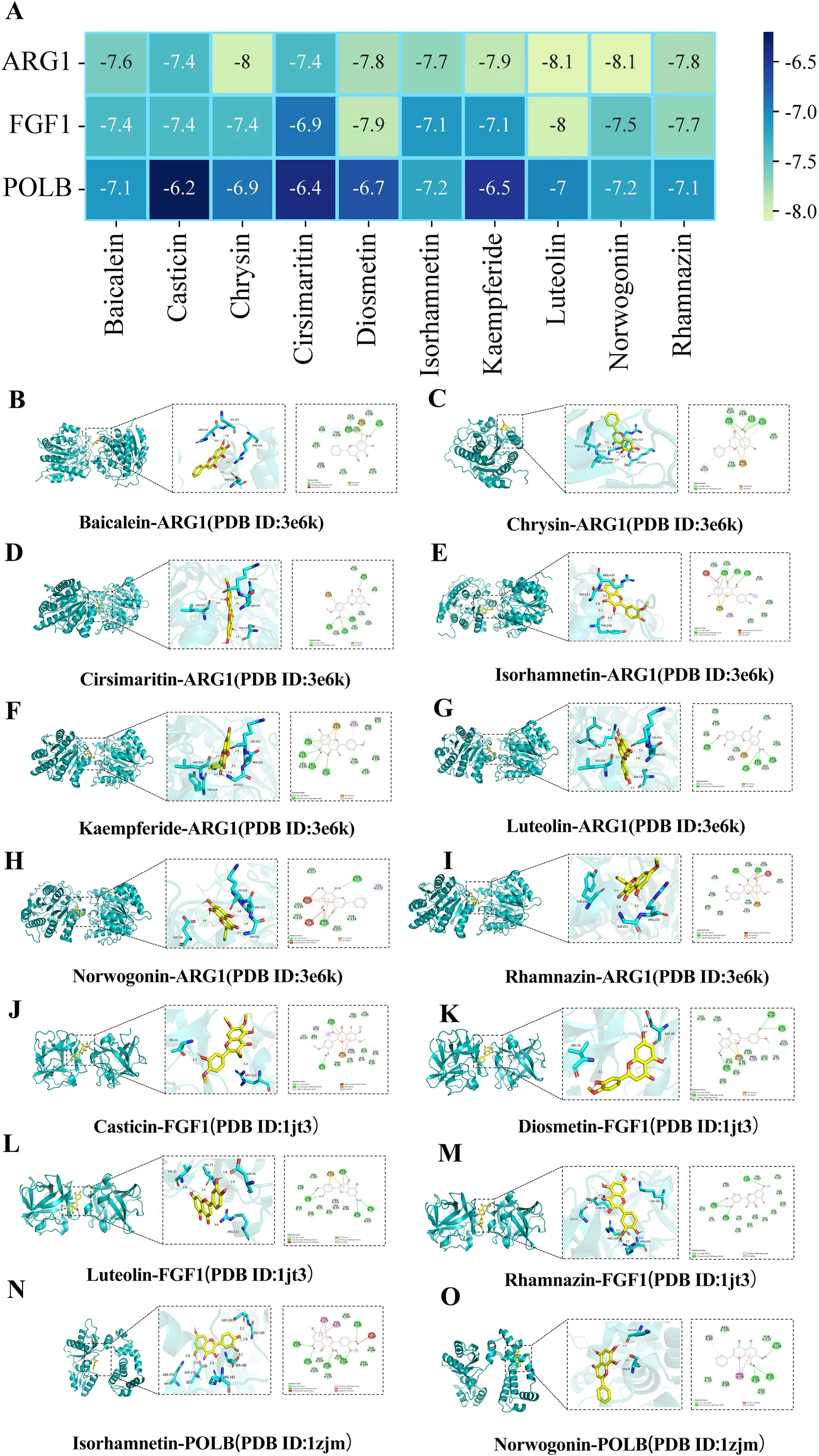
Molecular docking analysis of key components and core target of QFLTP. **(A)**Heat map of binding energies. **(B–O)** Molecular docking diagrams.

Molecular docking analysis was performed between the ten key bioactive compounds and the three hub proteins (POLB, FGF1, and ARG1). All tested compounds exhibited strong binding affinities (most docking scores were < −6.0 kcal/mol)(**Figure 5A**). Representative docking conformations visualized using PyMOL demonstrated stable ligand–protein interactions (**Figures 5B–O**).

#### 3.2.9 Molecular dynamics simulation

To evaluate the stability of the ligand–protein complexes, 100-ns molecular dynamics (MD) simulations were performed for the representative luteolin–ARG1 and norwogonin–ARG1 complexes. The simulations demonstrated that both complexes maintained stable conformations with persistent hydrogen-bond interactions throughout the simulation period, indicating stable and sustained ligand–target binding (**Supplementary Figures S1**, **2**).

### 3.3 Experimental validation

#### 3.3.1 General condition of mice

Relative to healthy baseline cohorts, murine subjects within the experimental disease group manifested prototypical clinical indicators characterizing ALI, including lethargy, reduced activity, tachypnea, respiratory distress, and increased secretions. These clinical manifestations indicated the successful induction of an acute pulmonary injury phenotype following LPS challenge. Treatment with dexamethasone or medium- and high-dose of QFLTP markedly improved general condition and activity levels.

#### 3.3.2 QFLTP alleviates lung tissue injury in ALI mice

**Figure 6:**
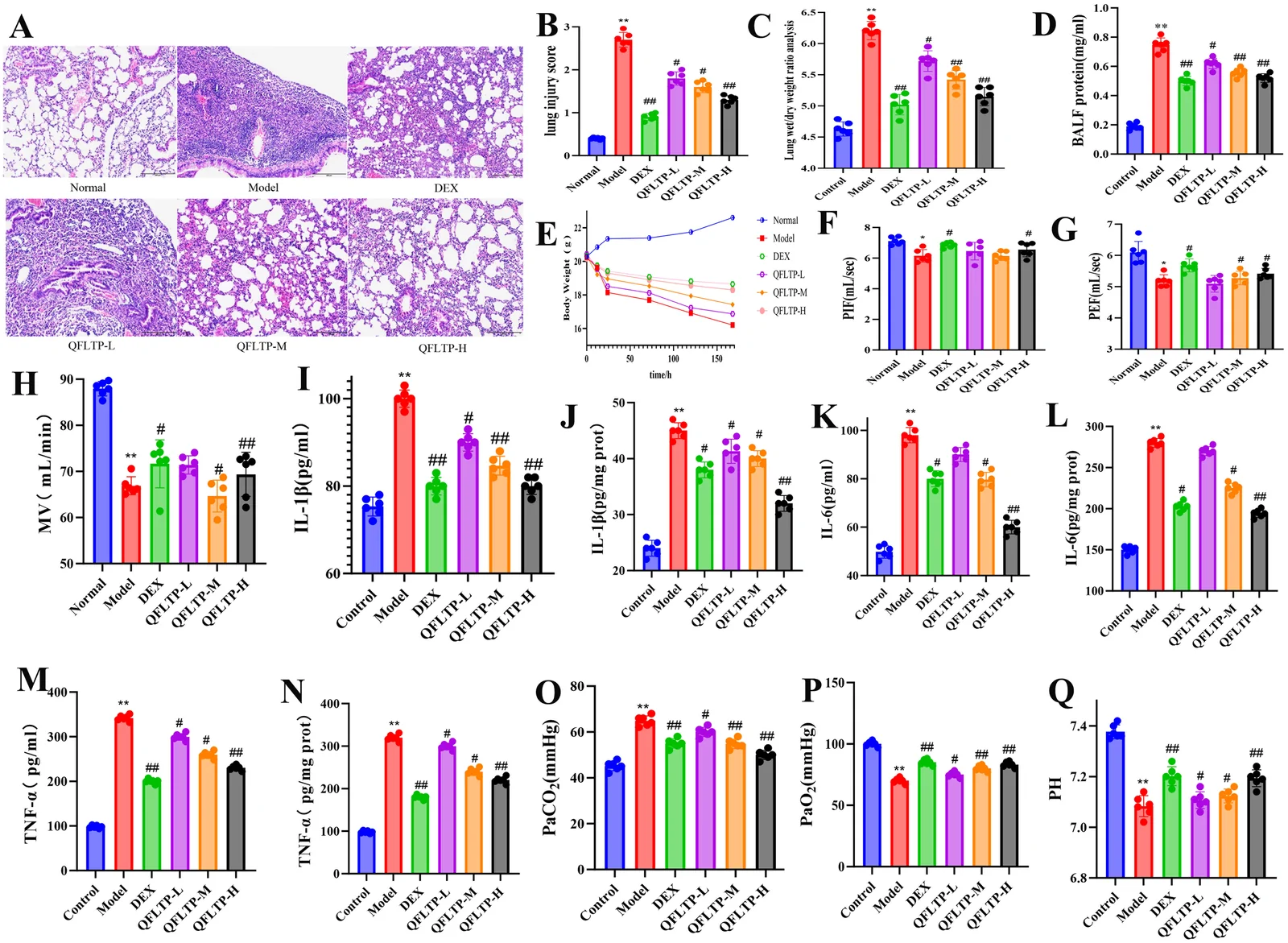
Therapeutic effects of QFLTP on LPS-induced ALI in mice. **(A)** Representative images of lung histopathological changes (H&E staining, ×200). **(B)** Lung injury score. **(C)** Lung wet/dry weight ratio. **(D)** BALF protein concentration. **(E)** Changes in body weight. **(F–H)** Changes in lung function parameters. **(I–N)** ELISA analysis of IL-1β, IL-6, and TNF-α levels in serum and lung tissues. **(O–Q)** Arterial blood gas analysis. Data are presented as mean ± SD (n = 6). ^*^P < 0.05, ^**^P < 0.01 compared with the Normal group; ^#^*P* < 0.05, ^##^*P* < 0.01 compared with the Model group.

Hematoxylin and eosin (H&E) staining demonstrated that QFLTP administration dose-dependently reduced inflammatory cell infiltration, alveolar septal thickening, and pulmonary tissue consolidation (**Figure 6A**). Quantitative analysis using the American Thoracic Society (ATS) lung injury scoring system showed significantly increased injury scores in the ALI model group compared with the control group, confirming the successful establishment of the LPS-induced ALI model, whereas QFLTP treatment significantly reduced lung injury scores in a dose-dependent manner. The high-dose QFLTP group exhibited a protective effect comparable to that of the dexamethasone group (**Figure 6B**).

#### 3.3.3 QFLTP attenuates pulmonary edema, weight loss, and inflammatory responses

To further validate the stability of the LPS-induced ALI model and evaluate the therapeutic effects of QFLTP, pulmonary edema, alveolar-capillary barrier integrity, respiratory function, and inflammatory responses were assessed. Compared with the control group, LPS administration significantly increased the lung wet/dry (W/D) weight ratio, indicating enhanced pulmonary fluid accumulation and pulmonary edema, which is a characteristic feature of ALI. Dexamethasone treatment markedly reduced the elevated W/D ratio, while QFLTP administration also significantly decreased pulmonary edema in a dose-dependent manner (**Figure 6C**). Consistently, LPS challenge resulted in a marked increase in BALF protein concentration, reflecting disruption of the alveolar-capillary barrier and increased pulmonary vascular permeability. QFLTP treatment significantly reduced BALF protein levels, suggesting restoration of barrier integrity (**Figure 6D**).In addition, QFLTP treatment attenuated body weight loss and improved pulmonary function parameters, including peak inspiratory flow (PIF), peak expiratory flow (PEF), and minute ventilation (MV), compared with the ALI model group (**Figures 6E–H**). Furthermore, QFLTP significantly decreased the serum and lung tissue levels of IL-1β, IL-6, and TNF-α, indicating that QFLTP effectively suppressed both systemic inflammation and local pulmonary inflammatory responses during ALI progression (**Figures 6I–N**). Arterial blood gas analysis further confirmed impaired pulmonary gas exchange after LPS exposure, as evidenced by altered oxygenation-related parameters, whereas QFLTP administration significantly improved oxygenation status and alleviated respiratory dysfunction in ALI mice (**Figures 6O–Q**).

#### 3.3.4 QFLTP restores endothelial and epithelial barrier integrity by upregulating junction proteins

**Figure 7:**
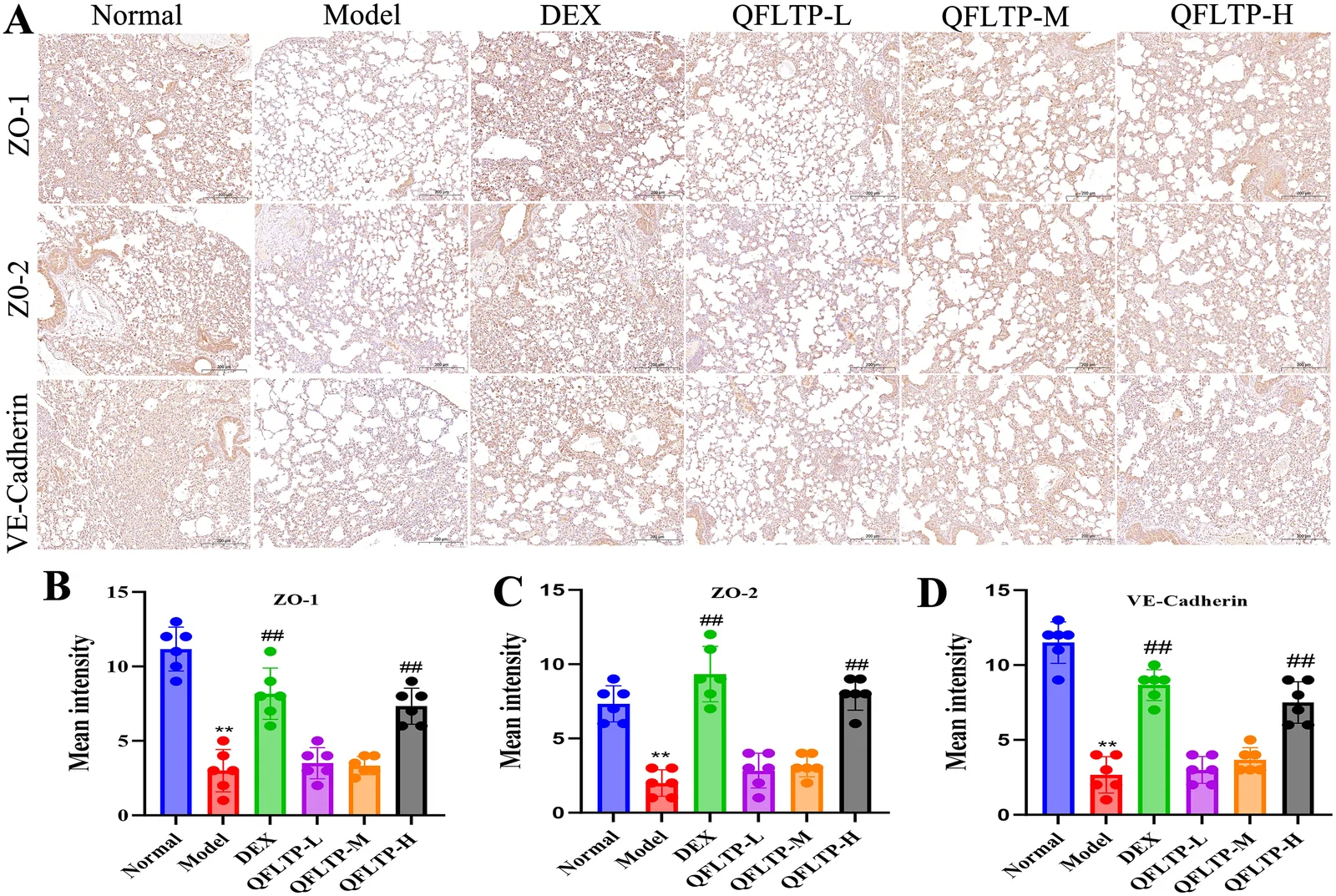
QFLTP upregulates the expression of junction proteins ZO-1, ZO-2, and VE-cadherin in lung tissues of LPS-induced ALI mice as detected by immunohistochemistry. **(A)** Representative immunohistochemical images of vascular endothelial-related proteins ZO-1, ZO-2, and VE-cadherin in lung tissues. **(B)** Statistical results of ZO-1 expression in the indicated groups. **(C)** Statistical results of ZO-2 expression in the indicated groups. **(D)** Statistical results of VE-cadherin expression in the indicated groups. Data are expressed as the mean ± SD (n = 6). ^*^P < 0.05,^**^P < 0.01 compared with the normal group; ^#^*P* < 0.05, ^##^*P* < 0.01 compared with the model group.

**Figure 8:**
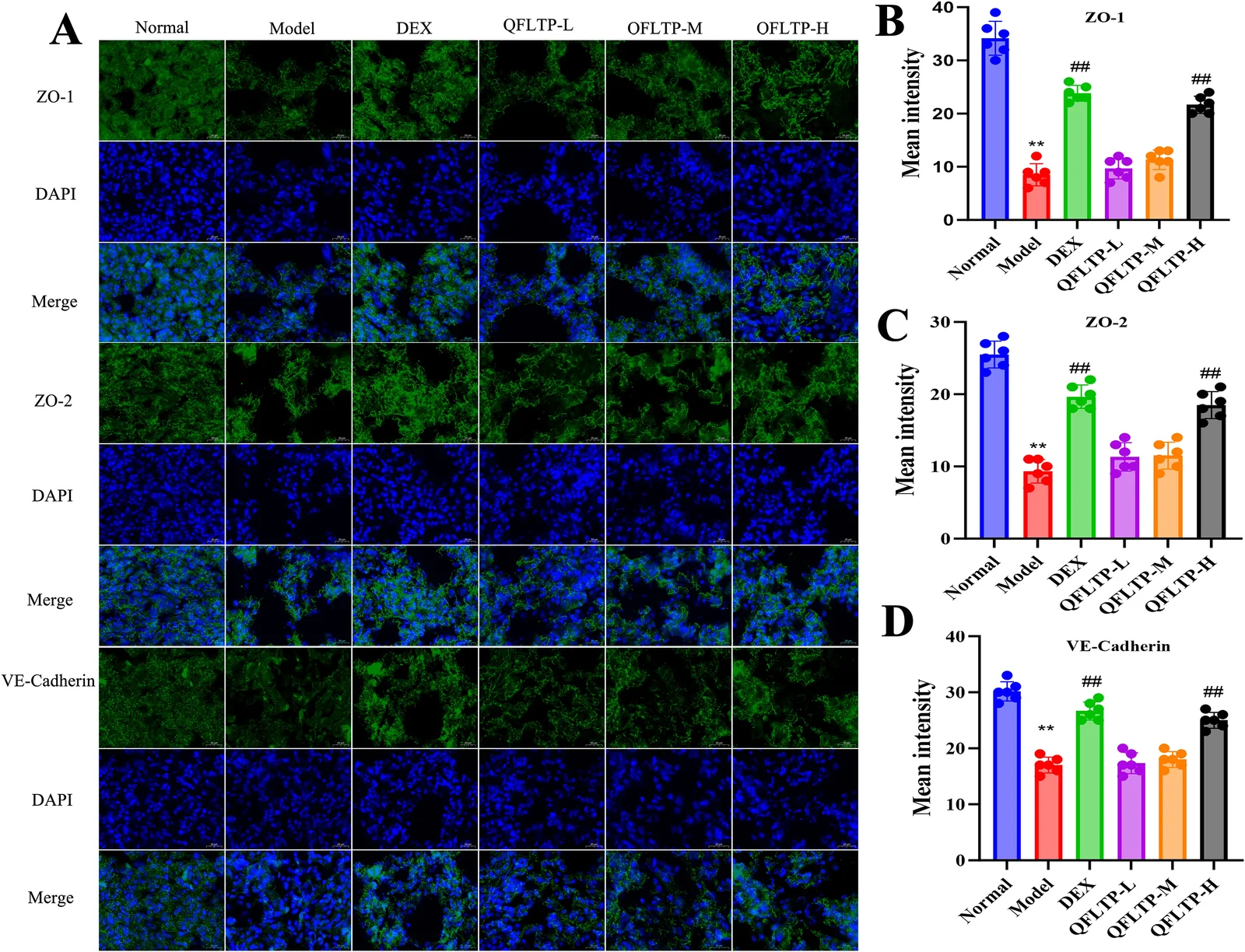
QFLTP restores the expression of junction proteins ZO-1, ZO-2, and VE-cadherin in lung tissues of LPS-induced ALI mice as detected by immunofluorescence. **(A)** Representative immunofluorescence images of vascular endothelial-related proteins ZO-1, ZO-2, and VE-cadherin in lung tissues. **(B)** Statistical results of ZO-1 expression in the indicated groups. **(C)** Statistical results of ZO-2 expression in the indicated groups. **(D)** Statistical results of VE-cadherin expression in the indicated groups. Data are expressed as the mean ± SD (n = 6). ^*^P < 0.05, ^**^P < 0.01 compared with the normal group; ^#^*P* < 0.05, ^##^*P* < 0.01 compared with the model group.

**Figure 9:**
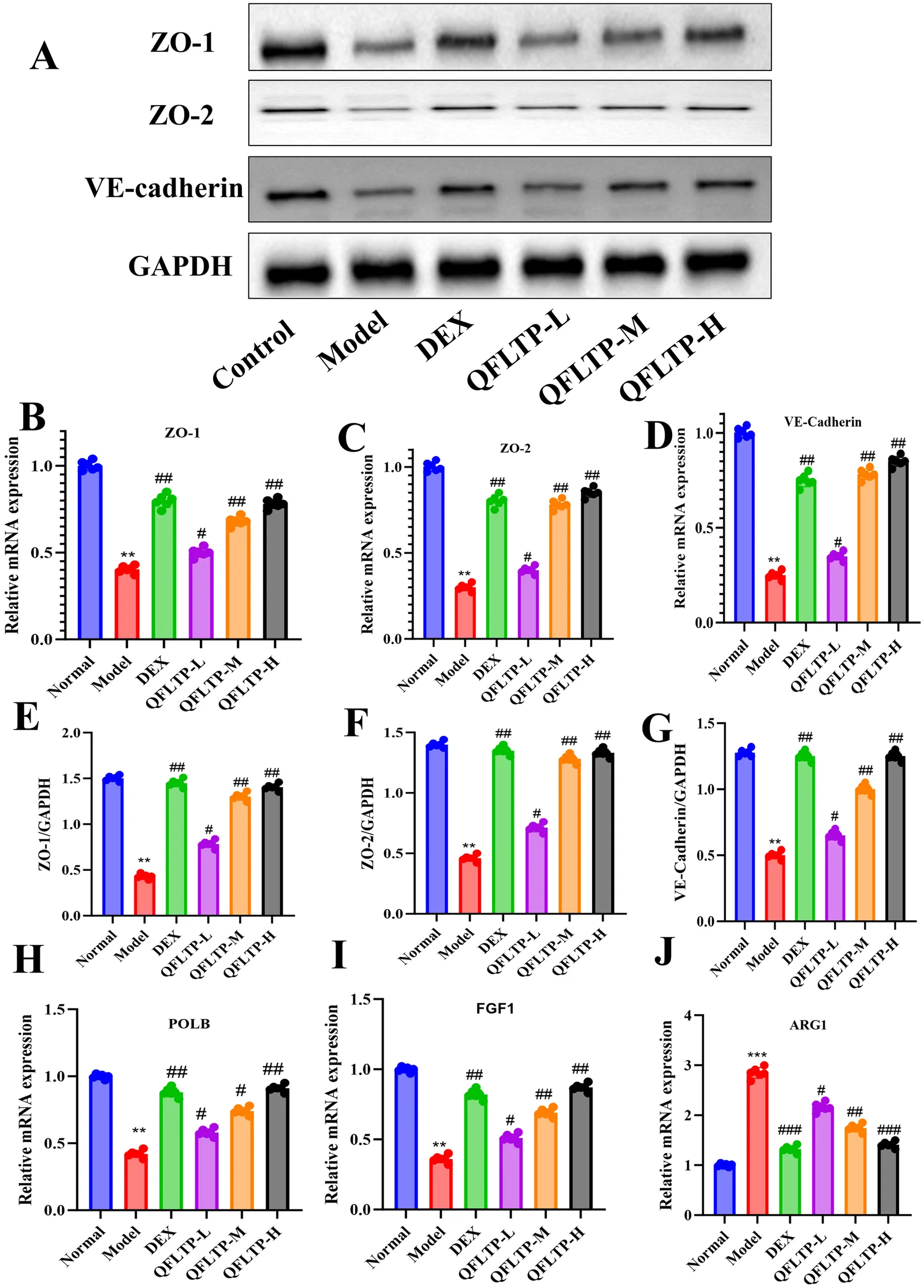
QFLTP regulates the expression of junctional proteins and hub genes in lung tissues of ALI mice. **(A)** Representative Western blot bands and quantitative analysis of VE-cadherin, ZO-2, and ZO-1 protein expression. **(B–D)** RNA expression levels of junctional proteins: **(B)** ZO-1, **(C)** ZO-2, and **(D)** VE-cadherin. **(E–G)** Relative quantitative expression of junctional proteins: **(E)** ZO-1, **(F)** ZO-2, and **(G)** VE-cadherin. **(H–J)** Relative mRNA expression of hub genes: **(H)** POLB, **(I)** FGF1, and **(J)** ARG1.Data are presented as mean ± SD (n = 6). ^*^P < 0.05, ^**^P < 0.01, ^***^P < 0.001 compared with the normal group; ^#^*P* < 0.05, ^##^*P* < 0.01, ^###^*P* < 0.001 compared with the model group.

Immunohistochemistry (**Figure 7**) and immunofluorescence (**Figure 8**) analyses demonstrated that QFLTP treatment, particularly at the high dose, significantly increased the expression levels of the tight junction proteins ZO-1, ZO-2, and VE-cadherin. Western blot analysis showed that the protein levels of ZO-1, ZO-2, and VE-cadherin were markedly decreased in the Model group compared with the Normal group, whereas QFLTP treatment dose-dependently restored their expression (**Figure 9A**). Consistent with the protein changes, qRT-PCR results demonstrated that the mRNA levels of ZO-1 (**Figure 9B**), ZO-2 (**Figure 9C**), and VE-cadherin (**Figure 9D**) were significantly downregulated in the Model group and were effectively upregulated by QFLTP. Quantitative densitometric analysis of the Western blot bands further confirmed that QFLTP significantly increased the relative protein expression of ZO-1 (**Figure 9E**), ZO-2 (**Figure 9F**), and VE-cadherin (**Figure 9G**) normalized to GAPDH.

#### 3.3.5 QFLTP regulates the expression of POLB, FGF1, and ARG1 in lung tissues

qRT-PCR analysis confirmed that QFLTP treatment significantly normalized the aberrant mRNA levels of the three hub genes in lung tissues of ALI mice. Specifically, the mRNA expression of POLB (**Figure 9H**) and FGF1 (**Figure 9I**) was markedly decreased, while ARG1 (**Figure 9J**) was significantly increased in the Model group. QFLTP administration effectively reversed these abnormal expression patterns in a dose-dependent manner, further supporting the involvement of POLB, FGF1, and ARG1 in the protective mechanism of QFLTP against ALI.

## 4 Discussion

ALI represents a major challenge in current clinical research. ALI takes hypoxemia as the main clinical manifestation, and the pathological changes are mainly diffuse interstitial destruction and alveolar edema. Numerous inflammatory mediators are produced by a significant number of inflammatory cells that have infiltrated and aggregated in lung tissues, which damage the alveolar-capillary barrier and seriously affect gas exchange in the lungs (Deng et al., 2020; Taylor et al., 2018). Sepsis caused by bacterial infection is associated with high mortality, and severe cases may progress to multiple organ dysfunction syndrome, with the lung being one of the most frequently affected organs. Currently, the epidemiological burden of ALI, including both morbidity and mortality, remains substantial, with mortality rates consistently exceeding 30% (Ahn et al., 2021; Butt et al., 2016; Jonas and Raj, 2020). Although ALI involves complex pathophysiological mechanisms, the precise mechanisms underlying the therapeutic effects of traditional Chinese medicine remain incompletely understood. QFLTP has demonstrated potential therapeutic effects in patients with pneumonia and LPS-induced ALI mice. However, the molecular mechanisms responsible for these effects remain unclear. To elucidate the protective mechanisms of QFLTP against ALI, this study integrated network pharmacology, bioinformatics analysis, molecular docking, molecular dynamics simulations, and *in vivo* validation using a mouse model. These findings provide new insights into potential therapeutic strategies for ALI and support the future clinical translation of QFLTP.

ALI progresses through three major pathological stages: the exudative, proliferative, and fibrotic phases. The typical pathological characteristics of ALI include alveolar wall thickening, capillary congestion, impaired pulmonary function, inflammatory cell infiltration, and pulmonary tissue consolidation. Among these pathological alterations, disruption of the alveolar-capillary barrier is a critical event contributing to pulmonary edema and impaired gas exchange during ALI progression. Pulmonary endothelial cells maintain vascular barrier homeostasis, whereas epithelial tight junctions preserve alveolar integrity. ZO-1 and ZO-2 are key tight junction proteins involved in maintaining epithelial barrier function, while VE-cadherin is an essential adhesion molecule regulating endothelial junction stability and vascular permeability (Balczon et al., 2024; Fliesser et al., 2023). The downregulation of these junction proteins increases vascular leakage, promotes inflammatory cell infiltration, and exacerbates pulmonary dysfunction; therefore, they are widely used as important indicators for evaluating barrier impairment in ALI (Vedpathak et al., 2023; Zeng et al., 2018).

To comprehensively evaluate the effects of QFLTP on pulmonary function, vascular permeability, pathological changes, and tight junction protein expression in ALI, we treated ALI mice with QFLTP and performed comprehensive experimental analyses. The animal experiments demonstrated that QFLTP treatment produced a dose-dependent protective effect in mice with LPS-induced ALI. The high-dose QFLTP group significantly improved lung histopathological changes, reduced inflammatory cell infiltration and alveolar edema, decreased systemic pro-inflammatory cytokine levels, improved pulmonary function and blood gas parameters, and increased the expression of VE-cadherin, ZO-1, and ZO-2. These findings suggest that QFLTP alleviates ALI by suppressing inflammatory responses and restoring alveolar-capillary barrier integrity.

In this study, UHPLC-MS/MS analysis identified 47 major chemical constituents in QFLTP. Building on this finding, we employed an “active ingredient–target–disease” strategy to identify the principal bioactive components associated with the therapeutic effects of QFLTP against ALI, including baicalein, casticin, chrysin, cirsimaritin, diosmetin, isorhamnetin, kaempferide, luteolin, norwogonin, and rhamnazin.

Baicalein, a prominent flavonoid compound isolated from Scutellaria baicalensis, possesses multiple pharmacological activities, including anti-inflammatory, anti-angiogenic, antitumor, antimicrobial, and antiviral effects. Jiang et al (Jiang et al., 2022). found that baicalein alleviates ALI by suppressing inflammation and restoring mitochondrial function. By inhibiting Drp1 expression, baicalein attenuates mitochondrial dysfunction and reduces excessive reactive oxygen species (ROS) production, thereby suppressing NF-κB-mediated inflammatory signaling. Consequently, the levels of TNF-α, MIP-1, and IL-6 are significantly reduced, accompanied by decreased neutrophil infiltration into lung tissues. These findings indicate that baicalein alleviates ALI by attenuating inflammatory responses and lung injury. Liu et al (Liu et al., 2025). further reported that baicalein protects against ALI by inhibiting the HIF-1α signaling pathway, thereby suppressing glycolysis, reducing inflammation and alveolar edema, and ultimately improving pulmonary function. Casticin, a major flavonoid isolated from *Vitex Fructus*, exhibits potent anti-inflammatory activity. Wang et al (Wang et al., 2016). demonstrated that casticin protects against ALI by inhibiting both the NF-κB signaling pathway and the NLRP3 inflammasome. Casticin markedly attenuated LPS-induced lung injury, as evidenced by reduced myeloperoxidase (MPO) activity and a lower lung wet/dry weight ratio. In addition, casticin significantly decreased the production of TNF-α, IL-6, and IL-1β through suppression of NF-κB activation and inhibition of NLRP3 inflammasome components, including ASC and caspase-1. Chrysin, a naturally occurring flavonoid, exerts significant protective effects against ALI by suppressing inflammatory responses and enhancing endogenous antioxidant defenses. In LPS-induced mouse models, chrysin significantly reduced lung injury scores, MPO activity, and the levels of pro-inflammatory cytokines in both lung tissues and BALF. It also alleviated pulmonary edema by reducing vascular permeability and enhancing the activities of antioxidant enzymes, including superoxide dismutase (SOD) and glutathione peroxidase (GSH-Px). These protective effects were mediated, at least in part, through inhibition of the IRE1α/TXNIP/NLRP3 signaling pathway (Chen et al., 2021). Cirsimaritin, a bioactive flavonoid isolated from Cirsium japonicum, has been reported to alleviate LPS-induced ALI (Lim et al., 2025). Its protective effects are associated with inhibition of NLRP3 inflammasome activation and downregulation of HIF-1α. Through modulation of these pathways, cirsimaritin suppresses the expression of pro-inflammatory mediators and glycolytic enzymes in macrophages, thereby reducing alveolar wall thickening, inflammatory cell infiltration, and hyaline membrane formation. Cell-based studies have shown that diosmetin promotes wound healing and enhances endothelial barrier function by upregulating ZO-1 and occludin expression in HUVECs. In addition, diosmetin reduces the expression of inflammatory cytokines, including TNF-α and IL-6, suggesting its potential as a natural therapeutic agent for ALI (Xia et al., 2023). Yang et al (Yang et al., 2022)reported that isorhamnetin alleviates ALI by inhibiting the TLR4/NF-κB pathway, reducing type II alveolar epithelial cell injury, promoting autophagy through mTOR inhibition, and decreasing epithelial cell apoptosis. Gao et al (Gao et al., 2024)found that kaempferide reduces sepsis-induced lung damage and inflammation by decreasing IL-6 and TNF-α, lowering endothelial permeability, inhibiting MLC2 hyperphosphorylation, and upregulating ZO-1, VE-cadherin, and occludin, thereby enhancing the lung endothelial barrier. Hou et al (Hou et al., 2022). demonstrated that luteolin alleviates ALI by promoting alveolar fluid clearance, reducing pulmonary edema, and enhancing epithelial sodium channel (ENaC) expression and activity through activation of the cGMP/PI3K signaling pathway. Cao et al (Cao et al., 2025)demonstrated that norwogonin alleviates ALI by regulating the Src/AKT1/NF-κB signaling pathway, thereby suppressing inflammation, reducing pulmonary edema, and restoring endothelial junction proteins. In addition, norwogonin directly binds to key targets, including AKT1, COX-2, and Src. Wu et al (Wu et al., 2017). reported that rhamnazin prevents ALI by activating the Nrf2-mediated antioxidant pathway, thereby reducing oxidative stress and inflammation. In conclusion, the major bioactive constituents of QFLTP exhibit considerable therapeutic potential against ALI through multiple complementary mechanisms, providing a promising multi-component, multi-target therapeutic strategy for ALI.

To build upon the identification of the key active constituents and their corresponding molecular targets, it is essential to further investigate the biological processes and signaling pathways underlying the therapeutic effects of QFLTP in ALI. Functional enrichment analysis provides a systematic approach for exploring these targets and elucidating the molecular mechanisms involved. These analyses help explain how multiple bioactive compounds synergistically regulate inflammatory responses, alleviate oxidative stress, and maintain cellular homeostasis.

GO enrichment analysis of the overlapping targets revealed significant enrichment in several biological processes, including macrophage colony-stimulating factor signaling, positive regulation of cell population proliferation, protein autophosphorylation, inflammatory response, peptidyl-tyrosine phosphorylation, negative regulation of apoptotic processes, responses to xenobiotic stimuli, and protein phosphorylation. These findings suggest that QFLTP alleviates ALI by suppressing inflammatory responses, promoting cytoprotection, and maintaining cellular homeostasis. Regarding cellular components, enrichment was primarily observed in membrane-associated structures, including dendrites, postsynapses, plasma membranes, and the extracellular space, suggesting that QFLTP may regulate membrane-associated signaling and intercellular communication. In terms of molecular functions, significant enrichment was observed in enzyme binding, protein tyrosine kinase activity, ATP binding, and protein kinase activity, indicating that QFLTP exerts its therapeutic effects by targeting key signaling enzymes and related catalytic pathways.

KEGG pathway analysis identified the PI3K-Akt and FoxO signaling pathways as major pathways involved in the therapeutic effects of QFLTP against ALI. These pathways play essential roles in regulating cell survival, inflammatory responses, oxidative stress, and immune homeostasis. Sun et al (Sun et al., 2024). demonstrated that Jingfang granules alleviate bleomycin-induced ALI by inhibiting the PI3K/Akt/mTOR signaling pathway, thereby improving glycolysis and pyruvate metabolism, reducing inflammation, and attenuating pulmonary fibrosis. Shi et al (Shi et al., 2019). further demonstrated that regulation of the PI3K/Akt pathway modulates HO-1 expression, thereby regulating mitochondrial fusion, fission, and mitophagy, ultimately reducing oxidative injury and improving ALI.

Subsequent GSEA was performed to characterize the distinct biological functions of these hub genes. POLB exhibited enrichment in pathways governing translational regulation, immune signaling, and metabolic adaptation, indicating its potential involvement in mitigating cellular stress and modulating apoptosis during the pathogenesis of ALI. Although POLB is traditionally recognized as a key DNA repair enzyme involved in base excision repair, increasing evidence suggests that DNA damage responses are closely associated with inflammation, oxidative stress, and cellular survival during tissue injury (Wu et al., 2018). Therefore, POLB may indirectly contribute to ALI progression by regulating cellular stress responses and maintaining genomic stability under inflammatory conditions. FGF1, as a canonical growth factor, was predominantly enriched in immune regulatory and autoimmunity-associated pathways, hinting at a bifunctional role in facilitating epithelial repair while preserving immune homeostasis during inflammatory stress (Dhlamini et al., 2022). Concurrently, ARG1 was linked to mucosal immunity, cellular adhesion, and antiviral defense mechanisms, underscoring its pivotal immunoregulatory contribution to the inflammatory microenvironment (Lucas et al., 2013).

The expression levels of these three hub genes were strongly associated with immune cell infiltration in the ALI microenvironment. ALI tissues displayed significant enrichment of activated B cells, dendritic cells, neutrophils, and CD8^+^ T cells, reflecting enhanced innate and adaptive immune activation, whereas regulatory and helper immune subsets were notably depleted. POLB exhibited positive correlations with activated B cells and certain immature immune populations, while FGF1 and ARG1 showed inverse relationships with immune activation markers, indicating their distinct regulatory roles in the immune response.

qRT-PCR analysis confirmed that QFLTP treatment effectively reversed the abnormal expression patterns of POLB, FGF1, and ARG1 in lung tissues. These changes were accompanied by improvements in immune cell infiltration patterns, suggesting that QFLTP suppresses excessive immune activation, promotes immune homeostasis, preserves pulmonary barrier integrity, and ultimately attenuates inflammatory lung injury.

Molecular docking was employed as a robust computational strategy to quantify the binding affinities and interaction modalities between bioactive small-molecule constituents and their putative protein targets. The top ten active compounds from QFLTP were docked against the core targets POLB, FGF1, and ARG1 previously identified through network pharmacology and machine learning. Most compounds formed stable complexes with favorable binding energies, typically ranging from -6.0 to below -7.0 kcal/mol, with several pairs demonstrating particularly strong binding affinities. These docking results strongly supported the predictions from network pharmacology and indicated that QFLTP may exert its protective effects by directly interacting with multiple key proteins involved in ALI pathogenesis. The four ligand-target pairs with the lowest binding energies were subsequently subjected to 100 ns molecular dynamics simulations, which demonstrated good stability of the complexes and persistent hydrogen bonding interactions.

This study has several limitations. First, only a limited number of hub genes were experimentally validated, and the complete molecular network underlying the therapeutic effects of QFLTP in ALI requires further investigation. Second, immune infiltration analysis was based on public transcriptomic data and should be validated by additional experimental approaches. Third, molecular docking and molecular dynamics simulations provide computational evidence of ligand–target interactions but cannot confirm direct binding under physiological conditions. Finally, only male mice were included in this study, and potential sex-related differences should be evaluated in future studies. Further multi-omics analyses and mechanistic experiments are needed to comprehensively elucidate the therapeutic mechanisms of QFLTP in ALI.

## 5 Conclusion

This study employed a multidimensional approach, integrating bioinformatics and experimental validation to elucidate the therapeutic mechanisms of QFLTP in the context of ALI. Through this methodology, three pivotal hub genes—POLB, FGF1, and ARG1—were identified as essential targets of QFLTP. The regulation of these genes is intimately linked to the restoration of pulmonary barrier integrity and the recalibration of systemic immune responses.

## Data Availability

The original contributions presented in the study are included in the article/**Supplementary Material**. Further inquiries can be directed to the corresponding authors.
