# Supplementary material for: Deciphering the therapeutic mechanisms of Qingfei Litan prescription in acute lung injury: an integrated framework of UHPLC–MS/MS, systems pharmacology, machine learning, and in vivo validation
Source: Front Cell Infect Microbiol. 2026 Sep 3;16:1898960. doi: 10.3389/fcimb.2026.1898960 (PMC13581640; doi:10.3389/fcimb.2026.1898960)
Supplement: Supplementary file 1 [file SupplementaryFile1.docx]

Supplementary Material

# 1 Supplementary Figures and Tables

For more information on Supplementary Material and for details on the different file types accepted, please see [here](https://www.frontiersin.org/guidelines/author-guidelines#supplementary-material).

## 1.1 Supplementary Figures

**Supplementary Figure 1.Molecular dynamics simulation results of the ARG1–Luteolin complex.**

**Supplementary Figure 2. Molecular dynamics simulation results of the ARG1–Norwogonin complex.**

**Supplementary Table S1.** The structure of the main components of QFLTP.

| NO | Component name | Molecular Formula | Structure | NO | Component name | Molecular Formula | Structure |
| --- | --- | --- | --- | --- | --- | --- | --- |
| 1 | Hopantenic acid | C_10_H_19_NO_5_ |  | **25** | Norwogonin | C_15_H_10_O_5_ |  |
| **2** | Cryptochlorogenic acid | [C](https://pubchem.ncbi.nlm.nih.gov/#query=C16H18O9)_[16](https://pubchem.ncbi.nlm.nih.gov/#query=C16H18O9)_[H](https://pubchem.ncbi.nlm.nih.gov/#query=C16H18O9)_[18](https://pubchem.ncbi.nlm.nih.gov/#query=C16H18O9)_[O](https://pubchem.ncbi.nlm.nih.gov/#query=C16H18O9)_[9](https://pubchem.ncbi.nlm.nih.gov/#query=C16H18O9)_ |  | **26** | Rhamnazin | C_17_H_14_O_7_ |  |
| **3** | Neoamygdalin | [C](https://pubchem.ncbi.nlm.nih.gov/#query=C20H27NO11)_[20](https://pubchem.ncbi.nlm.nih.gov/#query=C20H27NO11)_[H](https://pubchem.ncbi.nlm.nih.gov/#query=C20H27NO11)_[27](https://pubchem.ncbi.nlm.nih.gov/#query=C20H27NO11)_[NO](https://pubchem.ncbi.nlm.nih.gov/#query=C20H27NO11)_[11](https://pubchem.ncbi.nlm.nih.gov/#query=C20H27NO11)_ |  | **27** | Tricin | C_17_H_14_O_7_ |  |
| **4** | Amygdalin | [C](https://pubchem.ncbi.nlm.nih.gov/#query=C20H27NO11)_[20](https://pubchem.ncbi.nlm.nih.gov/#query=C20H27NO11)_[H](https://pubchem.ncbi.nlm.nih.gov/#query=C20H27NO11)_[27](https://pubchem.ncbi.nlm.nih.gov/#query=C20H27NO11)_[NO](https://pubchem.ncbi.nlm.nih.gov/#query=C20H27NO11)_[11](https://pubchem.ncbi.nlm.nih.gov/#query=C20H27NO11)_ |  | **28** | Baicalein | [C](https://pubchem.ncbi.nlm.nih.gov/#query=C15H10O5)_[15](https://pubchem.ncbi.nlm.nih.gov/#query=C15H10O5)_[H](https://pubchem.ncbi.nlm.nih.gov/#query=C15H10O5)_[10](https://pubchem.ncbi.nlm.nih.gov/#query=C15H10O5)_[O](https://pubchem.ncbi.nlm.nih.gov/#query=C15H10O5)_[5](https://pubchem.ncbi.nlm.nih.gov/#query=C15H10O5)_ |  |
| **5** | Taxifolin | [C](https://pubchem.ncbi.nlm.nih.gov/#query=C15H12O7)_[15](https://pubchem.ncbi.nlm.nih.gov/#query=C15H12O7)_[H](https://pubchem.ncbi.nlm.nih.gov/#query=C15H12O7)_[12](https://pubchem.ncbi.nlm.nih.gov/#query=C15H12O7)_[O](https://pubchem.ncbi.nlm.nih.gov/#query=C15H12O7)_[7](https://pubchem.ncbi.nlm.nih.gov/#query=C15H12O7)_ |  | **29** | Peiminine | [C](https://pubchem.ncbi.nlm.nih.gov/#query=C27H43NO3)_[27](https://pubchem.ncbi.nlm.nih.gov/#query=C27H43NO3)_[H](https://pubchem.ncbi.nlm.nih.gov/#query=C27H43NO3)_[43](https://pubchem.ncbi.nlm.nih.gov/#query=C27H43NO3)_[NO](https://pubchem.ncbi.nlm.nih.gov/#query=C27H43NO3)_[3](https://pubchem.ncbi.nlm.nih.gov/#query=C27H43NO3)_ |  |
| **6** | Prunasin | C_14_H_17_NO_6_ |  | **30** | Liquiritigenin | C_15_H_12_O_4_ |  |
| **7** | Isoliquiritin | C_21_H_22_O_9_ |  | **31** | Isoliquiritigenin | [C](https://pubchem.ncbi.nlm.nih.gov/#query=C15H12O4)_[15](https://pubchem.ncbi.nlm.nih.gov/#query=C15H12O4)_[H](https://pubchem.ncbi.nlm.nih.gov/#query=C15H12O4)_[12](https://pubchem.ncbi.nlm.nih.gov/#query=C15H12O4)_[O](https://pubchem.ncbi.nlm.nih.gov/#query=C15H12O4)_[4](https://pubchem.ncbi.nlm.nih.gov/#query=C15H12O4)_ |  |
| **8** | Liquiritin | C_21_H_22_O_9_ |  | **32** | Formononetin | [C](https://pubchem.ncbi.nlm.nih.gov/#query=C16H12O4)_[16](https://pubchem.ncbi.nlm.nih.gov/#query=C16H12O4)_[H](https://pubchem.ncbi.nlm.nih.gov/#query=C16H12O4)_[12](https://pubchem.ncbi.nlm.nih.gov/#query=C16H12O4)_[O](https://pubchem.ncbi.nlm.nih.gov/#query=C16H12O4)_[4](https://pubchem.ncbi.nlm.nih.gov/#query=C16H12O4)_ |  |
| **9** | Licraside | C_26_H_30_O_13_ |  | **33** | Pinellic Acid | C_18_H_34_O_5_ |  |
| **10** | Chrysin-6-C-Arabinoside 8-C-Glucoside | C_26_H_28_O_13_ |  | **34** | Tianshic Acid | C_18_H_34_O_5_ |  |
| **11** | Chrysin 6-C-glucoside-8-C-alpha-L-arabinopyranoside | C_26_H_28_O_13_ |  | **35** | Isorhamnetin | C_16_H_12_O_7_ |  |
| **12** | Luteolin | C_15_H_10_O_6_ |  | **36** | Isopeimine | C_27_H_45_NO_3_ |  |
| **13** | Quercitrin | C_21_H_20_O_11_ |  | **37** | Wogonin | [C](https://pubchem.ncbi.nlm.nih.gov/#query=C16H12O5)_[16](https://pubchem.ncbi.nlm.nih.gov/#query=C16H12O5)_[H](https://pubchem.ncbi.nlm.nih.gov/#query=C16H12O5)_[12](https://pubchem.ncbi.nlm.nih.gov/#query=C16H12O5)_[O](https://pubchem.ncbi.nlm.nih.gov/#query=C16H12O5)_[5](https://pubchem.ncbi.nlm.nih.gov/#query=C16H12O5)_ |  |
| **14** | Kaempferide 3-Glucoside | [C](https://pubchem.ncbi.nlm.nih.gov/#query=C22H22O11)_[22](https://pubchem.ncbi.nlm.nih.gov/#query=C22H22O11)_[H](https://pubchem.ncbi.nlm.nih.gov/#query=C22H22O11)_[22](https://pubchem.ncbi.nlm.nih.gov/#query=C22H22O11)_[O](https://pubchem.ncbi.nlm.nih.gov/#query=C22H22O11)_[11](https://pubchem.ncbi.nlm.nih.gov/#query=C22H22O11)_ |  | **38** | Chrysin | C_15_H_10_O_4_ |  |
| **15** | Complanatuside | C_28_H_32_O_16_ |  | **39** | Cirsimaritin | C_17_H_14_O_6_ |  |
| **16** | Licuroside | C_26_H_30_O_13_ |  | **40** | Casticin | [C](https://pubchem.ncbi.nlm.nih.gov/#query=C19H18O8)_[19](https://pubchem.ncbi.nlm.nih.gov/#query=C19H18O8)_[H](https://pubchem.ncbi.nlm.nih.gov/#query=C19H18O8)_[18](https://pubchem.ncbi.nlm.nih.gov/#query=C19H18O8)_[O](https://pubchem.ncbi.nlm.nih.gov/#query=C19H18O8)_[8](https://pubchem.ncbi.nlm.nih.gov/#query=C19H18O8)_ |  |
| **17** | Neoisoliquiritin | C_21_H_22_O_9_ |  | **41** | Acacetin | [C](https://pubchem.ncbi.nlm.nih.gov/#query=C16H12O5)_[16](https://pubchem.ncbi.nlm.nih.gov/#query=C16H12O5)_[H](https://pubchem.ncbi.nlm.nih.gov/#query=C16H12O5)_[12](https://pubchem.ncbi.nlm.nih.gov/#query=C16H12O5)_[O](https://pubchem.ncbi.nlm.nih.gov/#query=C16H12O5)_[5](https://pubchem.ncbi.nlm.nih.gov/#query=C16H12O5)_ |  |
| **18** | Baicalin | C_21_H_18_O_11_ |  | **42** | Kaempferide | C_16_H_12_O_6_ |  |
| **19** | Dihydrobaicalein 7-O-glucuronide | C_21_H_20_O_11_ |  | **43** | Sigmoidin B | [C](https://pubchem.ncbi.nlm.nih.gov/#query=C20H20O6)_[20](https://pubchem.ncbi.nlm.nih.gov/#query=C20H20O6)_[H](https://pubchem.ncbi.nlm.nih.gov/#query=C20H20O6)_[20](https://pubchem.ncbi.nlm.nih.gov/#query=C20H20O6)_[O](https://pubchem.ncbi.nlm.nih.gov/#query=C20H20O6)_[6](https://pubchem.ncbi.nlm.nih.gov/#query=C20H20O6)_ |  |
| **20** | Edpetiline | C_33_H_53_NO_8_ |  | **44** | Glycycoumarin | [C](https://pubchem.ncbi.nlm.nih.gov/#query=C21H20O6)_[21](https://pubchem.ncbi.nlm.nih.gov/#query=C21H20O6)_[H](https://pubchem.ncbi.nlm.nih.gov/#query=C21H20O6)_[20](https://pubchem.ncbi.nlm.nih.gov/#query=C21H20O6)_[O](https://pubchem.ncbi.nlm.nih.gov/#query=C21H20O6)_[6](https://pubchem.ncbi.nlm.nih.gov/#query=C21H20O6)_ |  |
| **21** | Wogonoside | [C](https://pubchem.ncbi.nlm.nih.gov/#query=C22H20O11)_[22](https://pubchem.ncbi.nlm.nih.gov/#query=C22H20O11)_[H](https://pubchem.ncbi.nlm.nih.gov/#query=C22H20O11)_[20](https://pubchem.ncbi.nlm.nih.gov/#query=C22H20O11)_[O](https://pubchem.ncbi.nlm.nih.gov/#query=C22H20O11)_[11](https://pubchem.ncbi.nlm.nih.gov/#query=C22H20O11)_ |  | **45** | Glyasperin C | [C](https://pubchem.ncbi.nlm.nih.gov/#query=C21H24O5)_[21](https://pubchem.ncbi.nlm.nih.gov/#query=C21H24O5)_[H](https://pubchem.ncbi.nlm.nih.gov/#query=C21H24O5)_[24](https://pubchem.ncbi.nlm.nih.gov/#query=C21H24O5)_[O](https://pubchem.ncbi.nlm.nih.gov/#query=C21H24O5)_[5](https://pubchem.ncbi.nlm.nih.gov/#query=C21H24O5)_ |  |
| **22** | Oroxylin A Glucoronide | C_22_H_20_O_11_ |  | **46** | Glyasperin F | [C](https://pubchem.ncbi.nlm.nih.gov/#query=C20H18O6)_[20](https://pubchem.ncbi.nlm.nih.gov/#query=C20H18O6)_[H](https://pubchem.ncbi.nlm.nih.gov/#query=C20H18O6)_[18](https://pubchem.ncbi.nlm.nih.gov/#query=C20H18O6)_[O](https://pubchem.ncbi.nlm.nih.gov/#query=C20H18O6)_[6](https://pubchem.ncbi.nlm.nih.gov/#query=C20H18O6)_ |  |
| **23** | Diosmetin | C_16_H_12_O_6_ |  | **47** | Licoisoflavone B | [C](https://pubchem.ncbi.nlm.nih.gov/#query=C20H16O6)_[20](https://pubchem.ncbi.nlm.nih.gov/#query=C20H16O6)_[H](https://pubchem.ncbi.nlm.nih.gov/#query=C20H16O6)_[16](https://pubchem.ncbi.nlm.nih.gov/#query=C20H16O6)_[O](https://pubchem.ncbi.nlm.nih.gov/#query=C20H16O6)_[6](https://pubchem.ncbi.nlm.nih.gov/#query=C20H16O6)_ |  |
| **24** | Peimine | C_27_H_45_NO_3_ |  |  |  |  |  |

**Supplementary Table S2.** The information of the primary KEGG pathways.

| Rank | Pathway | Genes | Count |
| --- | --- | --- | --- |
| 1 | Pathways in cancer | RET, ITGB1, ALK, GSK3B, HSP90AB1, FLT3, ITGA2B, PIK3CD, PIK3CB, FGF1, FGF2, IGF1R, IKBKB, EDNRA, RPS6KA5, CASP3, AKT2, AKT3, PIM1, AKT1, PDGFRB, PDGFRA, HSP90AA1, DAPK1, MMP1, MMP2, PRKCA, F2, MMP9, PGF, AR, PIK3CA, CCNE1, KIT, PPARG, RAF1, MET, PPARD, PTGER4, HDAC2, ROCK2, HDAC1, PTGER2, PTGER3, PIK3R1, PTGS2, EGFR, RELA, HSP90B1, RXRA, TERT, ABL1, MAPK3, NOS2, BRAF, ESR1, ESR2, NFKB1, IL2, PTK2, VEGFA, MAPK10, CDK6, RPS6KB1, CDK2, BCL2, MDM2, FGFR1, BCL2L1 | 69 |
| 2 | PI3K-Akt signaling pathway | RET, ITGB1, CHRM2, GSK3B, CHRM1, HSP90AB1, FLT3, ITGB3, ITGA2B, PIK3CD, PIK3CB, FGF1, FGF2, PIK3CG, IGF1R, IKBKB, AKT2, AKT3, KDR, AKT1, YWHAG, PDGFRB, PDGFRA, HSP90AA1, SYK, ITGA4, PDPK1, PRKCA, PGF, PIK3CA, CCNE1, KIT, RAF1, SGK1, MET, PIK3R1, EGFR, RELA, HSP90B1, RXRA, MCL1, MAPK3, NTRK2, INSR, NFKB1, IL2, PTK2, VEGFA, CDK6, RPS6KB1, CDK2, BCL2, MDM2, FGFR1, BCL2L1 | 55 |
| 3 | Prostate cancer | GSK3B, HSP90AB1, PIK3CD, PIK3CB, PIK3R1, RELA, EGFR, HSP90B1, IGF1R, IKBKB, PLAU, AKT2, AKT3, AKT1, MAPK3, PDGFRB, PDGFRA, HSP90AA1, PDPK1, MMP3, BRAF, MMP9, NFKB1, AR, PIK3CA, CCNE1, CDK2, BCL2, MDM2, RAF1, FGFR1 | 31 |
| 4 | EGFR tyrosine kinase inhibitor resistance | GSK3B, SRC, PIK3CD, PIK3CB, PIK3R1, FGF2, EGFR, IGF1R, AKT2, AKT3, KDR, AKT1, MAPK3, PDGFRB, PDGFRA, PRKCA, BRAF, VEGFA, PIK3CA, RPS6KB1, AXL, BCL2, RAF1, MET, BCL2L1 | 25 |
| 5 | Proteoglycans in cancer | ITGB1, ROCK2, SRC, ITGB3, PIK3CD, PIK3CB, PIK3R1, FGF2, TNF, EGFR, IGF1R, PLAU, CTSL, CASP3, AKT2, AKT3, KDR, AKT1, MAPK3, PDPK1, MMP2, PRKCA, BRAF, MAPK14, MMP9, ESR1, PTK2, VEGFA, PIK3CA, RPS6KB1, MDM2, PTPN6, HPSE, RAF1, MET, FGFR1 | 36 |
| 6 | Chemical carcinogenesis - receptor activation | HSP90AB1, SRC, CHRNA7, PIK3CD, ADRB1, AHR, ADRB2, PIK3CB, PIK3R1, CYP3A4, FGF2, RELA, EGFR, HSP90B1, RPS6KA3, RXRA, AKT2, AKT3, CYP1B1, AKT1, MAPK3, HSP90AA1, EPHX2, PRKCA, ESR1, ESR2, NFKB1, VEGFA, AR, PIK3CA, RPS6KB1, CYP1A2, CYP1A1, BCL2, RAF1, PPARA | 36 |
| 7 | Ras signaling pathway | PLA2G1B, FLT3, PIK3CD, PLA2G5, PIK3CB, PIK3R1, FGF1, FGF2, RELA, EGFR, IGF1R, IKBKB, AKT2, AKT3, KDR, ABL1, AKT1, MAPK3, PDGFRB, PDGFRA, NTRK2, INSR, PLA2G2A, PLA2G4A, PRKCA, PGF, NFKB1, VEGFA, MAPK10, ZAP70, PIK3CA, PLA2G10, KIT, RAF1, MET, FGFR1, BCL2L1 | 37 |
| 8 | Central carbon metabolism in cancer | PDGFRB, RET, PDGFRA, G6PD, FLT3, PIK3CD, PIK3CB, PIK3R1, EGFR, HK2, HK1, PIK3CA, AKT2, KIT, AKT3, AKT1, RAF1, MET, PDK1, FGFR1, MAPK3 | 21 |
| 9 | Lipid and atherosclerosis | GSK3B, HSP90AB1, ROCK2, SRC, PIK3CD, PIK3CB, PIK3R1, TNF, RELA, HSP90B1, IKBKB, RXRA, CASP3, AKT2, AKT3, AKT1, MAPK3, HSP90AA1, MMP1, PDPK1, MMP3, PRKCA, MAPK14, MMP9, PTK2, NFKB1, ERN1, MAPK10, CYP2C9, PIK3CA, CYP1A1, BCL2, PPARG, BCL2L1 | 34 |
| 10 | Platelet activation | ITGB1, ROCK2, SRC, ITGB3, ITGA2B, PIK3CD, PIK3CB, PIK3R1, PIK3CG, MYLK, PTGS1, TBXA2R, AKT2, AKT3, AKT1, FYN, PRKG1, MAPK3, PTGIR, SYK, PLA2G4A, MAPK14, F2, PIK3CA, TBXAS1, BTK | 26 |
| 11 | Rap1 signaling pathway | ITGB1, SRC, ITGB3, ITGA2B, PIK3CD, PIK3CB, PIK3R1, FGF1, FGF2, EGFR, IGF1R, CNR1, AKT2, AKT3, KDR, AKT1, DRD2, MAPK3, PDGFRB, PDGFRA, INSR, PRKCA, BRAF, MAPK14, PGF, VEGFA, ADORA2A, PIK3CA, ADORA2B, KIT, RAF1, MET, FGFR1 | 33 |
| 12 | Focal adhesion | ITGB1, GSK3B, ROCK2, SRC, ITGB3, ITGA2B, PIK3CD, PIK3CB, PIK3R1, EGFR, IGF1R, MYLK, AKT2, AKT3, KDR, AKT1, FYN, MAPK3, PDGFRB, PDGFRA, ITGA4, PDPK1, PRKCA, BRAF, PGF, PTK2, VEGFA, MAPK10, PIK3CA, BCL2, RAF1, MET | 32 |
| 13 | Endocrine resistance | SRC, MMP2, PIK3CD, BRAF, PIK3CB, PIK3R1, MAPK14, ESR1, MMP9, EGFR, PTK2, ESR2, IGF1R, MAPK10, PIK3CA, RPS6KB1, AKT2, AKT3, MDM2, BCL2, AKT1, RAF1, MAPK3 | 23 |
| 14 | PD-L1 expression and PD-1 checkpoint pathway in cancer | ALK, CSNK2A1, PIK3CD, PIK3CB, PIK3R1, MAPK14, EGFR, RELA, NFKB1, IKBKB, ZAP70, PIK3CA, RPS6KB1, LCK, AKT2, AKT3, TLR9, AKT1, PTPN6, PRKCQ, RAF1, MAPK3 | 22 |
| 15 | AGE-RAGE signaling pathway in diabetic complications | MMP2, PRKCD, SERPINE1, PIK3CD, PRKCA, PIK3CB, PIK3R1, MAPK14, F3, TNF, RELA, NFKB1, VEGFA, MAPK10, PIK3CA, CASP3, AKT2, AKT3, PIM1, BCL2, NOX4, AKT1, MAPK3 | 23 |
